# Influence of oxygen concentration on the metabolism of *Penicillium chrysogenum*

**DOI:** 10.1002/elsc.202100139

**Published:** 2022-04-07

**Authors:** Agnes Janoska, Jelle J. Verheijen, Wenjung Tang, Queenie Lee, Baukje Sikkema, Walter M. van Gulik

**Affiliations:** ^1^ Department of Biotechnology Delft University of Technology Delft The Netherlands; ^2^ DSM Biotechnology Center Alexander Fleminglaan 1 Delft Netherlands

**Keywords:** black box model, metabolic modeling, oxygen limitation, penicillin pathway, scale‐down

## Abstract

In large‐scale bioreactors, there is often insufficient mixing and as a consequence, cells experience uneven substrate and oxygen levels that influence product formation. In this study, the influence of dissolved oxygen (DO) gradients on the primary and secondary metabolism of a high producing industrial strain of *Penicillium chrysogenum* was investigated. Within a wide range of DO concentrations, obtained under chemostat conditions, we observed different responses from *P. chrysogenum*: (i) no influence on growth or penicillin production (>0.025 mmol L^−1^); (ii) reduced penicillin production, but no growth limitation (0.013–0.025 mmol L^−1^); and (iii) growth and penicillin production limitations (<0.013 mmol L^−1^). In addition, scale down experiments were performed by oscillating the DO concentration in the bioreactor. We found that during DO oscillation, the penicillin production rate decreased below the value observed when a constant DO equal to the average oscillating DO value was used. To understand and predict the influence of oxygen levels on primary metabolism and penicillin production, we developed a black box model that was linked to a detailed kinetic model of the penicillin pathway. The model simulations represented the experimental data during the step experiments; however, during the oscillation experiments the predictions deviated, indicating the involvement of the central metabolism in penicillin production.

**Abbreviations**
DO: dissolved oxygen (concentration)
CFD: computational fluid dynamics
MFC: mass flow controller
q_i_
: biomass specific production/consumption rates of compound i (e.g., oxygen, carbon dioxide, sugar, and penicillin)
C_i_
: concentration of compount i
μ: growth rate
ACV: δ‐(L‐α‐aminoadipoyl)‐L‐cysteinyl‐D‐valine
bisACV: bis‐δ‐(L‐α‐aminoadipoyl)‐L‐cysteinyl‐D‐valine
IPN: isopenicillin N
PAA: phenylacetic acid
oOHPAA: o‐hydroxyphenylacetic acid
6APA: 6‐aminopenicillanicacid
8HPA: 8‐hydroxypenicillic acid
OPC: 6‐oxopiperidine‐2‐carboxylic acid
PhAc‐CoA: phenylacetyl‐CoA
PIO: penicilloic acid
PenG: penicillin‐G
AAA: L‐α‐amino adipate
Val: valine
Cys: cysteine
ACVS: δ‐(L‐α‐aminoadipoyl)‐L‐cysteinyl‐D‐valine syntetase
IPNS: isopenicillin‐N synthetase
AAT: acyl‐CoA: 6APA acyltransferase
IAT: isopenicillin‐N acyltransferase
IAH: isopenicillin‐N amido‐hydrolase
PAH: phenylacetate hydroxylase

## 1 INTRODUCTION

In large‐scale aerobic fermentation processes, sufficient oxygen supply IS often a challenge, because the mixing and the mass transfer is poor, especially when high biomass densities are reached and/or when the broth is highly viscous. To supply sufficient oxygen, intense mixing required could harm the cells. In industrial scale bioreactors, dissolved oxygen (DO) concentration wide range variations can occur, both in axial and radial directions in the reactor space [1, 2, 3, 4]. As the cells travel through the reactor, they experience these spatially varying DO levels as fluctuations in oxygen concentration [5]. These differences in oxygen concentration have a significant impact on the metabolic response of the cells, affecting their metabolism and product formation [6]. The cell lifelines are the trajectories of individual cells in response to substrate and DO concentrations in the large‐scale bioreactor, which can be described with computational fluid dynamics (CFD) models.

A scale‐down approach is usually used to study the effects of environmental variations present in large‐scale bioreactors [5, 7]. Using this approach, the cell lifelines in large‐scale systems are simulated in lab‐scale bioreactors, preventing the need for experimenting on a large scale. For the scale‐down design, lifelines are divided into metabolic regimes, and by determining the residence times in each regime, rational scale‐down designs can be achieved [8]. This approach is useful for optimizing bioprocesses on a lab scale, which can improve large‐scale production by optimizing strains or bioreactor configurations. This scale‐down approach has been applied in multiple bioprocesses with a wide range of microorganisms [9, 10, 11, 12].

**PRACTICAL APPLICATION**

The biological understanding of the *Penicillium chrysogenum* response to limiting dissolved oxygen (DO) conditions is essential for optimized large‐scale penicillin production, where there is limited oxygen transfer that contributes to heterogeneous oxygen concentrations. Revealing the metabolic responses of the cells to low and fluctuating DO conditions provides two strategies for optimizing penicillin production. Firstly, metabolic engineering could be applied: in this study we showed that penicillin production was limited by the isopenicillin‐N synthetase (IPNS) enzyme under steady low DO conditions, while in oscillating DO conditions, the central metabolism was limited and increased ATP/precursor availability might promote production. Secondly, the design of a large‐scale penicillin production reactor could be improved to limit oxygen gradients, for example, by introducing additional sparger points. Finally, the developed model can be coupled to a computational fluid dynamics (CFD) model to predict the cellular growth and penicillin production on a seconds scale in the bioreactor under different operational conditions.

The DO concentration is a crucial parameter in the penicillin fermentation process. In addition to the demand of *Penicillium chrysogenum* cells for oxygen for growth and maintenance, molecular oxygen is also required in the product biosynthesis pathway. The second enzyme of penicillin production pathway, isopenicillin‐N synthetase (IPNS), requires O_2_ for the conversion of δ‐(L‐α‐aminoadipoyl)‐L‐cysteinyl‐D‐valine (ACV) to isopenicillin N (IPN) [13], which is further converted to penicillin‐G. Therefore, low oxygen levels affect both the primary and secondary metabolism of *P. chrysogenum* cells. However, the oxygen concentration that limits the oxygen uptake rate of cells is different from the oxygen concentration that limits penicillin production [14]. Vardar and Lilly reported that oxygen uptake is below approximately 0.019 mmol L^−1^ DO, while penicillin production sharply decreases at 0.082 mmol L^−1^ DO [14]. Similarly, Henriksen et al. also reported that at 0.019 mmol L^−1^ DO, cellular respiration and growth was unaffected, while the penicillin production decreased to zero [15]. These studies show that penicillin production stops below a certain DO level [14, 15]. Nevertheless, there is still questions regarding the reversibility of penicillin production after exposure to low DO conditions [14, 15]. The reversibility of the loss of penicillin productivity and the central metabolism might be related to exposure time and minimum DO levels, while the reversibility of the respiratory capacity was reported to be dependent on the growth phase [16, 17]. Experiments conducted under steady, low DO levels showed that the pathway metabolite levels change at low DO conditions [15]. The δ‐(L‐α‐aminoadipoyl)‐L‐cysteinyl‐D‐valine (ACV) levels increased at low DO because its enzymatic conversion to IPN was limited; however, the measured IPN concentrations did not match the expectations of IPNS enzyme inhibition [15]. These findings show that the mechanism through which low oxygen levels affect the penicillin pathway is still not completely understood.

Scale‐down studies of *P. chrysogenum* that used periodic oxygen limitations have been conducted in one compartment vessels with intermittent feeding regimes [14] or multiple vessels [18]. The study by Larsson et al. [18] focused on the influence of the DO concentration on respiratory activity rather than penicillin production. They found that after 2 h of short DO cycling that involved 1–2 min in an anaerobic compartment that accounted for 1% of the culture volume, the respiratory capacity was restored; while during longer cycles of 5–10 min in the anaerobic compartment that accounted for 6 % of the culture volume, there was irreversible inhibition of the respiration [18]. Vardar and Lilly [14] reported that the penicillin production rate was reduced by fluctuating DO levels between 0.063 and 0.010 mmol L^−1^, compared to the penicillin production rate at a steady DO level of the fluctuation average (0.082 mmol L^−1^). In contrast, during oscillation cycles between 0.027 and 0.041 mmol L^−1^, the penicillin production rate was higher than during a steady DO value of 0.034 mmol L^−1^. According to our knowledge, no studies have investigated the metabolite concentrations of the penicillin pathway and the connected primary metabolism under scaled down conditions with oscillating DO levels, which could provide information that could lead to deeper understanding of these observations.

Understanding the cellular responses to a fluctuating environment and describing those by kinetic models can lead to the identification of metabolic engineering targets for improved productivity [19]. When these kinetic models are coupled to hydrodynamic simulations, an accurate process description is achieved, where environmental variations on timescales of seconds can be evaluated [9, 19]. The resulting integrated model can be applied for process optimization, which next to strain improvements, can also assist design improvement and evaluation [9, 20].

Most modeling studies that describe the effect of DO on the penicillin pathway [21, 22] rely on the finding from Bainbridge [23], which showed that the purified IPNS enzyme has a first order dependence on oxygen between 0.068 and 0.191 mmol L^−1^ DO. Modeling aspects on penicillin production are currently lacking solid evidence for the effect of DO levels below 0.068 mmol L^−1^, and the assumed linearity might not be valid in a broader DO ranges. A detailed understanding of the influence of DO concentrations on the penicillin pathway is required to predict the penicillin production rate under dynamic conditions and develop dynamic models that can be coupled to CFD simulations.

In this study we aim to (i) understand correlations between DO levels and primary and secondary metabolic processes and (ii) model the influence of low and oscillating DO conditions on penicillin fermentation. We expand our existing knowledge on the influence of DO concentration on penicillin production and central metabolism using oxygen levels in the growth‐limiting range, which have not been studied in detail. For this, stimulus‐response experiments and scale‐down experiments are conducted, which provide powerful insight into the in vivo kinetics of this system [20]. Additionally, metabolome analysis was performed to investigate the cellular mechanisms that respond to environmental perturbations. To interpret the data and predict changes in the cellular metabolism, a black box model was built to represent the central metabolism of the cells. This model was coupled to a detailed enzyme kinetic model of the penicillin pathway, which considered the oxygen limitation of the IPNS enzyme and thus, on penicillin production. The integrated model considers the limitation of oxygen uptake and penicillin production rates at low oxygen concentrations, as well as takes into account the repressing effect of high sugar concentrations on the penicillin gene cluster [24]. Increased residual sugar concentrations are expected in oxygen limited zones because under oxygen limited conditions, the sugar uptake rate is expected to decrease. Increased sugar concentrations may lead to penicillin gene cluster inhibition and alter the outcome of the kinetic model. The obtained model is a simple interpretation of the influence oxygen has on the central metabolism and on penicillin biosynthesis, which can be directly used in combination with CFD [8, 25, 26], or can be a precedent of a detailed structured dynamic metabolic model [27, 28].

## 2 MATERIALS AND METHODS

### 2.1 Strain and inoculation

A high‐yielding, penicillin producing *Penicillium chrysogenum* strain (DS17690) donated by Centrient Pharmaceuticals (Delft, The Netherlands) as spores grown on rice grains was used in all experiments. Two different reactor configurations with total volumes of 2 and 7 L and corresponding working volumes of 1.25 and 4 L were inoculated with 3.1 or 10.0 g of rice, respectively. The spores were separated from the rice grains by stirring for at least 1 h in 50 and 100 mL sterile demineralized water. These solutions were filtered through a mesh to remove the rice and the resulting spore suspension was aseptically transferred to the 2 and 7 L reactors, respectively.

### 2.2 Medium composition

The *P. chrysogenum* cultivation medium was prepared as previously described [29]. The cultivation medium for the chemostat operation contained 5 g L^−1^ (NH_4_)_2_SO_4_, 1 g L^−1^ KH_2_PO_4_, 0.5 g L^−1^ MgSO_4_⋅7H_2_O, 16.5 g L^−1^ C_6_H_12_O_6_⋅H_2_O, 0.68 g L^−1^ phenylacetic acid (PAA) and 2 mL L^−1^ trace element solution. The trace element solution contained 75 g L^−1^ Na_2_EDTA⋅2H_2_O, 10 g L^−1^ ZnSO_4_⋅7H_2_O, 10 g L^−1^ MnSO_4_⋅1H_2_O, 20 g L^−1^ FeSO_4_⋅7H_2_O, 2.5 g L^−1^ CaCl_2_⋅2H_2_O, 2.5 g L^−1^ CuSO_4_⋅5H_2_O. The cultivation medium was used for the batch phase, except the PAA concentration was lowered to 0.41 g L^−1^. The PAA concentration in the batch medium (3 mM) was lower than in the chemostat medium (5 mM) in order to keep the residual PAA level within a minimal range of variation [30]. However, for the experiments in the 2 L reactor, 5 mM PAA was used for both batch and chemostat phases.

The trace elements were dissolved in demineralized water as two separate stocks (MnSO_4_⋅H_2_O and FeSO_4_⋅7H_2_O separated from the rest), which were then mixed and the pH was adjusted to 6.0 using NaOH. The solution was stirred overnight and stored at 4°C until needed.

The salts, glucose, and trace elements were dissolved in demineralized water, and the pH was adjusted to 5.5 with KOH. The PAA was dissolved separately in demineralized water with a PAA:KOH molar ratio of 1:1.2, and then the pH was adjusted to 5.5 with KOH or H_2_SO_4_. The total amount of PAA required for the media was dissolved in 4%–10% of the total volume. The PAA solution was autoclaved at 121°C. The medium without the PAA was filter‐sterilized (Sartopore MidiCaps 0.2 μm filters) and added to the autoclaved PAA solution.

### 2.3 Bioreactor setup

All experiments were conducted in stirred tank bioreactors (Applikon, Delft, The Netherlands), which were stirred with magnetically driven six‐bladed Rushton impellers. The details of the experimental setups and fermentation conditions are presented in Table 1.

**TABLE 1:** Experimental conditions used in this study

	Reference experiment	DO Step 0.025 Mm	DO Step 0.013 mM	DO Step 0.009 mM	Oscillation I	Oscillation II
Total volume (L)	2	7	2	7	7	7
Working volume (L)	1.25	4	1.25	4	4	4
Pressure (bar)	1	1.3	1.3	1.3	1.3	1.3
Number of stirrers (‐)	1	2	1	1	1	2
Stirrer diameter (mm)	45	85	45	85	85	85
Agitation speed (rpm)	600–800^a^	500	700	365	365	500
Total gas flow (L min^−1^)	0.41	2	0.41	2	2	1 and 4^b^
pH control	2 M KOH	4 M NaOH	2 M KOH	2 M KOH	2 M KOH	4 M NaOH
DO probes^c^	1, C	1, C	2, C&O	2, C&C/O	2, C	2, C&C/O
Frequency of antifoam addition	1 drop/3 h	1 drop/1.7 h	1 drop/3 h	1 drop/1.7 h	1 drop/1.7 h	1 drop/1.7 h
Batch phase media PAA concentration	5 mM	3 mM	5 mM	3 mM	3 mM	3 mM
Effluent	Overflow tube	Pneumatic valve+pump	Overflow tube	Pneumatic valve+pump	Pneumatic valve+pump	Pneumatic valve+pump
DO control, MFC controller configuration	Configuration ‘A’	Configuration ‘B’	Configuration ‘B’	Configuration ‘B’	Configuration ‘A’	Configuration ‘C’

^a^ During the chemostat phase of the reference experiments, the stirrer speed was between 600 and 800 rpm in order to keep the DO above 0.136 mmol L^−1^.

^b^ In the oscillation II experiment, the airflow was shifted from 1 L min^−1^ during the chemostat to 4 L min^−1^ in the oscillation phase.

^c^ ‘‘C’’ indicates the conventional and ‘O’ the optical DO probe. Where ‘C/O’ is indicated, a conventional or optical probe was used in one of duplicate fermentation experiments.

The pH, temperature (T), and DO were continuously measured and logged during the fermentations. The pH was measured (Mettler Toledo 405‐DPAS‐SC‐K8S) and maintained at a value of 6.50 ± 0.05 with an automatic pH control system, that supplied a base solution (KOH or NaOH) by a peristaltic pump. The applied base type is not expected to have any impact on the fermentations. The temperature was maintained at 25°C by a thermo‐circulator that controlled the water temperature based on the measured temperature of the reactor content.

During the DO step down experiments, the DO was controlled based on measurement by a conventional Clark electrode (Applikon Z01002325) or an optical DO probe (Hamilton Visiferm ECS 225 H0, USA) to keep the DO at the desired level. During the reference experiments, air was supplied to the reactor and the DO was not controlled.

**FIGURE 1:**
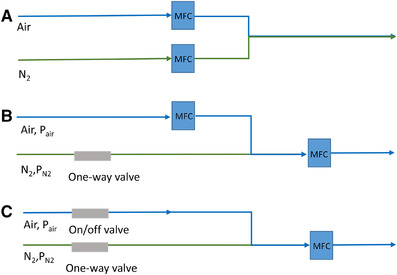
Gas supply setup to the bioreactor. (A) Reference experiment and oscillation experiment I. In the reference experiments, the N_2_ flow was closed. (B) Step down experiments to 0.013 mmol L^−1^ and 0.009 mmol L^−1^ DO, respectively. During the step experiment to 0.025 mmol L^−1^, opposing configuration was used where N_2_ had a higher pressure than air, which was controlled by MFCs, while the total flow was controlled by another MFC. (C) Oscillation experiment II. The on/off valve either allowed or blocked air flow into the system. In most experiments, a one‐way valve controlled the gas stream towards the reactor and not towards the MFC

In oscillation experiment I, two separate mass flow controllers (MFCs) provided either air or N_2_ as inlet gas to the reactor. These MFCs were controlled based on the measurements from a DO probe, and the switches between the MFCs and thus, between the inlet gases took place at given DO levels (Figure 1A). During the 0.009 and 0.013 mmol L^−1^ DO step experiments, the DO of the broth was maintained by controlling the composition of the aeration gas, which was based on the measured DO in the reactor. The aeration gas was composed of a mixture of air and N_2_. Air was supplied through an MFC at a higher pressure compared to the N_2_ flow, and the desired total flow rate was controlled by a second MFC. Therefore, the total gas flow was kept constant while the oxygen content of the aeration gas was adjusted by controlling the air flow rate (Figure 1B). In oscillation experiment II, the inlet gas was switched between air and N_2_ based on given time intervals with an on/off valve on the air line. When the valve was open, only air entered the reactor because it was provided at a higher pressure then N_2_; when the valve was closed, N_2_ gas entered the reactor at the same flow rate as air by the MFC. Air was provided for 30 s and N_2_ for 90 s, respectively; resulting in 120 s cycles. The exact procedure of DO control of this experiment is presented in Figure 1C.

The 7 L reactor was placed on a load cell, enabling continuous monitoring of broth weight. During chemostat cultivation, the dilution rate of all experiments was kept constant at 0.05 h^−1^ by setting the medium inflow rate. While the inflow was supplied continuously by a peristaltic pump, the effluent was removed periodically by an automatically controlled pneumatic valve on the bottom of the reactor and a peristaltic pump to regulate reactor weight. In this way, the broth volume was controlled at 4000 ± 20 g. The effluent from the 2 L reactor was removed every 30 min via an overflow tube. The feed medium vessels were continuously stirred. The effluent vessel was placed on a load cell that continuously recorded its weight to determine the exact dilution rate. The weight of the base solution and antifoam was also recorded. The overpressure of the reactor headspace was set by a reducing valve in the gas outlet. The reactors were aerated by a sparger underneath the impeller. The inlet gas flow was controlled by mass flow controllers (Brooks instrument B.V., The Netherlands). Before the gas entered the reactor, it was passed through a sterile 0.2 μm membrane filter (Millipore). The offgas left the reactor via a condenser at 4°C, through a sterile cotton wool filter. The O_2_ and CO_2_ concentrations in the offgas were quantified with a gas analyzer (Rosemount NGA 2000, USA). An approximate drop of antifoam (Basildon Chemical Co. Ltd, UK, Foam‐clear EscaFerm S) was added to the system by switching on the antifoam pump for a few seconds at given time intervals (see Table 1). The T, pH, stirrer speed, mass flows, offgas concentration, antifoam addition, reactor weight, and DO (if applicable) were controlled and/or logged by a DCU3 measurement and control unit (Sartorius, Germany). These parameters were logged on a minute scale; however, during the DO oscillation experiments, more frequent data logging on a seconds scale was used in order to identify changes of the logged parameters within the first and last oscillation cycle.

### 2.4 Experimental design

All fermentation experiments were started with a batch phase that lasted approximately 60 h, after which continuous operation was initiated. The pH control and antifoam addition began after the spores had germinated, which took approximately one day. Thereafter, to avoid splashing or depositing spores on the lid and glass wall above the liquid, which would result in wall growth, the airflow and stirrer speed were increased stepwise until they reached their final values. The chemostat phase was initiated after the glucose of the batch medium was exhausted, which was apparent from a sharp increase of DO, a sharp decrease of respiratory activity, and an increase of a pH above 6.5. In each experiment, air was supplied to the reactor and the DO was not controlled for approximately 5 residence times (100 h). During this phase, the DO remained at values between 0.136 and 0.190 mmol L^−1^. After reaching a steady‐state, the DO perturbation was initiated, where reduced or oscillating DO was applied. In the step experiments, the DO was reduced to 0.025, 0.013, or 0.009 mmol L^−1^ by supplying a mixture of air and N_2_. In the oscillation experiments I and II, air and N_2_ were supplied alternately to the reactor (see detailed description above).

During the oscillation experiments I and II, the average DO in a cycle and the cycle time remained similar for both experiments, with an average DO of 0.062 and 0.058 mmol L^−1^ after correction for probe delay and an average cycle time of 112 s and 120 s, respectively. The two experiments differed in the amplitude of the oscillations, whereby the DO in oscillation I varied in a smaller range (0 – 0.127 mmol L^−1^) compared to oscillation II (0 – 0.178 mmol L^−1^). Furthermore, in oscillation I, the DO fell below 0.003 mmol L^−1^ for 11 s, while in oscillation II, it was below 0.003 mmol L^−1^ for 23 s. Therefore, in oscillation II, the cells were exposed to higher DO variations and longer periods of near‐zero DO levels. The DO during a cycle in each oscillation experiment is presented in the Supplementary Material B, Figure B12. During the oscillation I experiment, the samples during the oscillation phase were exposed to the highest DO value of the DO cycle, with the first and last DO oscillation cycles sampled at ∼40 s intervals for intracellular metabolite analysis; while during oscillation experiment II, only the last cycle was sampled at more frequent time intervals.

The recovery of the cells and the penicillin production rates were investigated after the 0.009 and 0.013 mmol L^−1^ steps and in oscillation experiment I by increasing the DO after 5 residence times back to non‐limiting conditions that were above 0.136 mmol L^−1^.

### 2.5 Sampling and sample analysis

#### 2.5.1 Sampling for biomass dry weight, total organic carbon, and HPLC analysis

The biomass concentration was determined with dry weight measurements of culture samples withdrawn from the reactor. Samples were taken with a 60 mL sampling bottle attached to the reactor, where the broth entered due to a pressure difference, while in some cases a syringe was used to help creating vacuum in the bottle. Aliquots of 5 mL culture broth were collected in triplicate and filtered through dried and pre‐weighed glass fiber filters (Pall, type A/E 47 mm, 1 μm pore size). These filters were dried for at least 12 h at 70°C and then cooled in a desiccator for 1 h before pre‐weighing. Filtration was done with a vacuum pump and the filtrate was stored at ‒80°C until it was used for HPLC and total organic carbon (TOC) analysis. After collecting the filtrate, the mycelium on the filter was washed twice with 10 mL demineralized water, dried at 70°C for at least 24 h and then cooled in a desiccator for 1 h before weighing.

#### 2.5.2 Rapid sampling and sample processing for intracellular metabolite quantification

Rapid sampling and quenching was performed with a dedicated rapid sampling device [31]. Right before sampling, the broth was circulated in a 8 mm internal diameter tube with a peristaltic pump and from this loop approximately 1 mL of fermentation broth was withdrawn rapidly using a rapid sampling device that worked similarly to principles described by Lameiras et al. [31]. The broth was sampled after 0.13 s travelling time through the tubing. The sample was quenched immediately in 8 mL 40 % methanol and precooled to ‒25°C [32]. The exact volume of the sample was determined by weighing the methanol tubes before and after sampling. The quenched solution was then filtered through a glass fibre filter (Pall, USA, type A/E 47 mm, 1 μm pore size) [29], which was pre‐cooled with 20 mL ‒25°C 40 % methanol. The cells were washed twice with 20 mL and 40 mL 40 % methanol at ‒25°C. Next, the filter was transferred to 25 mL 75 % ethanol at 75°C and adjusted to pH 10.6 with 2 M KOH as described previously [31]. The pH adjustments were done in order to enable NADH analysis because NADH degrades at neutral pH [33]. The tubes also contained 500 μL 10 mM maleimide solution to enable quantification of the free reduced form of ACV [34]. Simultaneously with adding the filter to the heated ethanol tube, 100 μL 13 C pen extract was added as internal standard for the MS analysis. The tube was heated at 95°C for 3 min with shaking in a water bath and then cooled on ice for at least 5 min. The samples were stored at ‒80°C until further processing. Next, the thawed samples were vortexed, the filters were removed and the ethanol was evaporated using a RapidVap vacuum evaporation system (LABCONCO, USA) at 30°C and 40 % speed. The process continued until the sample volume decreased to ∼300 μL [31, 35], which took approximately 2–3 h. The evaporated sample was transferred to an Eppendorf tube and brought to a final volume of 500 μL with milli‐Q water. The samples were then centrifuged with 16,000 × *g* for 15 min (Heraeus Biofuge Pico, Germany). The supernatant was collected and stored at ‒80°C until analysis.

#### 2.5.3 Sampling for extracellular metabolite quantification

By using the rapid sampling device, approximately 1 mL broth was withdrawn and immediately quenched by cooling to approximately 1°C within a precooled syringe at ‒20°C that contained stainless steel beads (8.8 g, 4 mm diameter) [36]. Immediate quenching is required to avoid further glucose consumption and the conversion of extracellular glucose polymers, such as trehalose, to glucose [37]. Next, the cooled sample was filtered through a syringe filter (0.45 μm millex‐HV filter Millex, USA) to remove any biomass before a 100 μL aliquot of the filtrate was transferred to an Eppendorf tube containing 20 μL ^13^C labeled yeast extract stored on ice for metabolite quantification with isotope dilution mass spectrometry (IDMS). The sample mixed with the labelled cell extract and the remaining 900 μL of sample were immediately frozen in liquid nitrogen and stored at ‒80°C until analysis. A 100 μL aliquot was removed from the thawed 900 μL sample and transferred to a tube containing 20 μL ^13^C labelled *P. chrysogenum* cell extract. Extracellular glucose and trehalose concentrations were determined from the samples containing yeast extract, while the extracellular metabolites of the penicillin pathway were analyzed from the samples mixed with *P. chrysogenum* extract.

#### 2.5.4 Analytical procedures

Penicillin G and phenylacetic acid concentrations in the culture filtrate were quantified with high‐performance liquid chromatography (HPLC) using a Zorbax SB‐C18, 4.6 × 12.5 mm, 5 μm, guard column and a Zorbax SB‐C18, 4.6 × 75 mm, 3.5 μm analytical column (Agilent, USA) kept at 25°C. The eluent contained 5 mM KH_2_PO_4_ in 28 % acetonitrile dissolved in water, pH 2.5 or 3 with 85 % phosphoric acid; the flowrate was 1 mL min^−1^. The defrosted samples were stored in the autosampler of the HPLC at 4°C (Waters 2695, USA). The quantification was performed with a photodiode array detector at 214 nm (Waters 996, USA).

Both the intracellular penicillin metabolites and amino acids were analyzed from the samples taken from the rapid sampling device and processed by cold methanol quenching, while the extracellular metabolites of the penicillin pathway were analyzed from samples obtained from the rapid sampling device and quenched by the cold beads, as explained in Sections 2.5.2 and 2.5.3, respectively. The intra‐ and extracellular metabolites of the penicillin pathway were analyzed by ion‐pair reversed‐phase liquid chromatography‐isotope dilution electrospray ionization tandem mass spectrometry (IP–LC–ESI–ID–MS/MS) [38], where ^13^C‐labeled *Penicillium chrysogenum* cell extract was used as an internal standard. The intracellular amino acids were analysed by GC‐MS as described by de Jonge et al. [37].

When the extracellular glucose concentration was higher than 200 μM, HPLC analysis was used to quantify the glucose concentration of the broth. The eluent (100× diluted 85 % phosphoric acid, boiled to remove gas bubbles) had a flow rate of 0.6 mL min^−1^ and the run time was 30 min. The autosampler (Waters 717, USA) and analytical ion‐exchange column (Aminex HPX‐87, Bio‐Rad, Hercules, CA, USA) were maintained at 60°C; a UV detector (Waters 2489, USA) and a refraction index detector (Waters 2414, USA) were also used for the analysis of samples. For glucose concentrations below 200 μM, glucose and trehalose were quantified using GC‐MS as previously described [39]. For both methods, the samples were withdrawn with the rapid sampling device and quenched with cold steel beads, as explained in Section 2.5.3.

The total organic carbon concentration was quantified by subtracting the measured amount of inorganic carbon from the measured total amount of carbon present in the sample using a TOC analyzer (TOC‐L CSH, Shimadzu, Japan). Microscopic analysis (Carl Zeiss, Germany) was conducted using 15× diluted biomass samples at 10× and 100× magnification.

### 2.6 Calculations and data processing

#### 2.6.1 DO probe delay

The DO values reported in this study were converted from the DO probe measurements and expressed as % air saturation at atmospheric pressure to a concentration in mmol L^−1^ by applying the Henry constant of 1.3 × 10^−5^ mol m^−3^ Pa^−1^ [40], resulting in a solubility of 0.272 mmol L^−1^. During the oscillation experiments, the time delay of the DO probe response [41] was especially important when DO changes on a scale of seconds. To calculate the actual DO from the probe signal, the probes were assumed to have a first order response [41, 42]. The probe response time was measured by placing the probe from a stirred vessel saturated with N_2_ gas into a stirred vessel saturated with air. The probe delay was determined from the exponential curve fitted to the measured DO response. With the measured probe time constant, τ_probe_, the ‘‘real’’ DO C_O2,L_ was than calculated using Equation 1, where the differential term was obtained from the finite differences of the measured DO (C_O2,L_^M^).

(1) $$C_{O_{2},L}=C_{O_{2},L}^{M}+\tau_{\mathrm{probe}}\frac{{\mathrm{dC}}_{O_{2},L}^{M}}{dt}$$

After the corrections, the curve was smoothed by taking a 9‐s moving average and negative DO values were corrected to 0 mmol L^−1^ DO.

#### 2.6.2 Biomass specific conversion rates

To obtain the biomass specific rates under non‐steady state conditions, a polynomial curve was fitted to the experimental concentration data and from the derivative of the fitted polynomial, the infinitesimal changes in time were estimated. The q_O2_ and q_CO2_ rates were calculated assuming quasi steady state, as the DO stabilised rapidly and the gas and liquid phase accumulation terms were considered to be negligible compared to the uptake rates. In this way, from the measured biomass, residual sugar, and penicillin concentrations and the offgas composition, the biomass specific growth, penicillin production, and oxygen uptake/carbon dioxide emission rates were calculated using the corresponding material balances.

#### 2.6.3 Model solvers

A system of differential equations describing the kinetics of the enzymatic conversion steps and the changes in extra‐ and intracellular metabolite concentrations based on mass balances was set up and solved in Matlab 2018b. The details of the solver and experimental data processing are presented in the Appendix.

## 3 RESULTS

### 3.1 Short DO‐perturbation experiments

**FIGURE 2:**
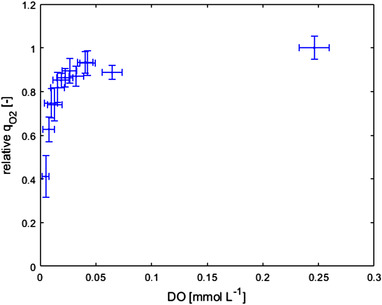
Relative biomass specific oxygen uptake rate normalized to the q_O2_ of the non‐limiting condition during the short DO – perturbation experiments

In order to determine the specific oxygen uptake (q_O2_) and carbon dioxide production (q_CO2_) rates in response to low DO concentrations, a glucose limited chemostat experiment at a dilution rate of 0.05 h^−1^ was performed. This experiment aimed to reveal the DO values that affected q_O2_, and was further used in model developed to estimate the half‐saturation constant for the cell oxygen uptake rates. After a steady state was reached, the DO was reduced in several steps to values ranging between 0.246 and 0.005 mmol L^−1^. Each step had a duration of 2 h, after which the DO was increased back to air saturation level for the subsequent 3 h. During cultivation, degeneration took place and therefore, penicillin production slowly declined over time [43]. The decreased q_p_ under sugar limitation resulted in an increased C_x_ and decreased biomass specific sugar and oxygen uptake rates. To account for this gradual loss of productivity and change in q_O2_ in these chemostat experiments, a relative oxygen uptake rate was measured, where the oxygen uptake rate during the step down was normalized to the oxygen uptake rate at non‐limiting DO levels just before the step. The relative rates were multiplied by the q_O2_ before degeneration started to obtain the corrected q_O2_. These results showed that below a DO of 0.025 mmol L^−1^, a sharp reduction in the q_O2_ occurred (Figure 2).

### 3.2 Long‐term oxygen step down experiments

**FIGURE 3:**
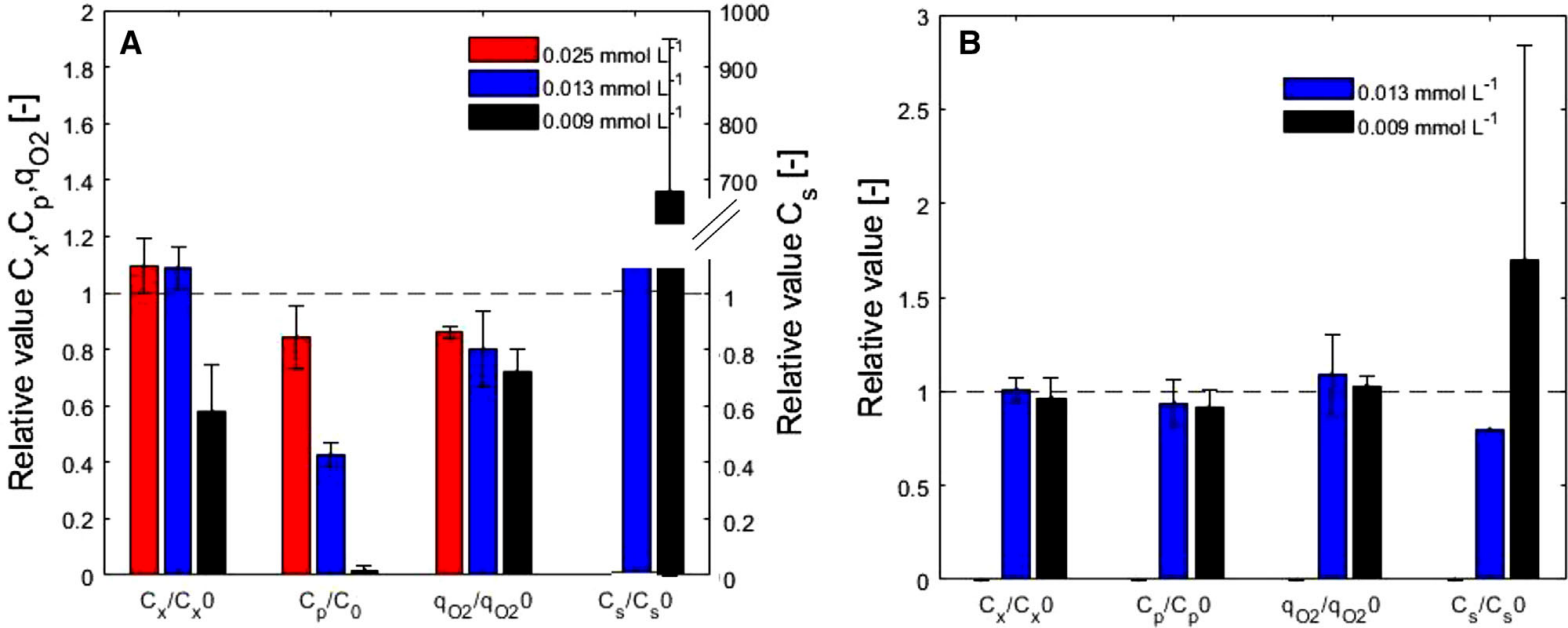
Relative C_x_, C_p_, q_O2_ and C_s_ values of the three different step experiments compared to the initial steady state condition. (A) At the end of the step‐down phase (∼100 h after step‐down) and (B) after the step‐down phase, the DO was restored to non‐limiting values (∼45 h after the DO was increased)

In a series of steady‐state chemostat cultures, the influence of DO concentrations of 0.009, 0.013, 0.025, or >0.136 mmol L^−1^ on the frementation process was investigated. These step experiments aimed to reveal the long‐term metabolic responses of *P. chrysogenum* cells to low DO values, including intra‐ and extracellular penicillin pathway metabolite levels. The measured penicillin concentrations were also used in simulations that estimated the parameter K_o_^IPNS^ (see Section 3.4). The results were evaluated in terms of changes in the measured biomass specific oxygen uptake rate (q_O2_), and concentrations of biomass (C_x_), sugar (C_s_), and penicillin (C_p_) relative to the values during the initial chemostat phase and at the end of the low DO step (Figure 3). The actual measurements of C_x_, C_s_, and C_p_ in the four step experiments are shown in Supplementary Material A and Figure A1. The morphological observations are presented in Supplementary Material A and Figure A5.

#### 3.2.1 Biomass specific conversion rates

During the steady states before the steps, the biomass concentrations were between 5.2 and 6.6 g L^−1^ and dependent on the reactor set‐up, which was consistent with the reported values of approximately 5.7 g L^−1^ for the same strain grown under similar chemostat conditions [37]. The biomass concentration and the calculated specific growth rates were conversely affected by low DO conditions during the step experiments. At DO levels of 0.013 mmol L^−1^ and 0.025 mmol L^−1^, we observed a slight increase in C_x_, while at 0.009 mmol L^−1^ DO, the biomass concentration decreased (Figure 3A) and the growth rate decreased to 0.045 h^−1^. The reduction in C_x_ was accompanied by an increase in C_s_ because the growth rate had decreased to a value lower than the dilution rate and therefore, the supplied sugar was not completely consumed.

The concentration of by‐products in the culture was quantified as the difference between the measured total organic carbon concentration (TOC) in the culture supernatant and the sum of the residual sugar, penicillin, and PAA concentrations. The by‐product formation rate was only affected by a 0.009 mmol L^−1^ DO, at which the by‐product concentration doubled or tripled; this translates to a biomass specific by‐product formation rate increase of five to eight times compared to the steady state value (data not shown).

**FIGURE 4:**
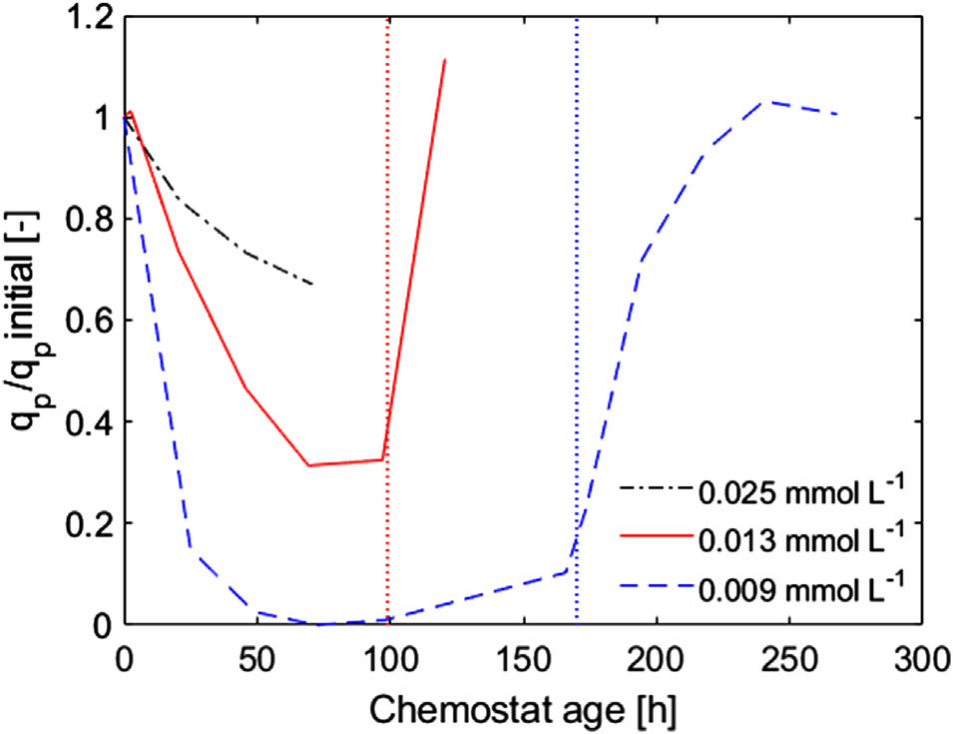
Calculated relative penicillin production rate during the step experiments. The q_p_ values were normalized to the initial steady state at non‐limiting DO levels (> 0.136 mmol L^−1^). Time point zero represents the start of the step‐down while the vertical dotted lines represent end of the step‐down phase, where the DO was restored to non‐limiting values. The first line corresponds to the 0.013 mmol L^−1^ step, while the second one to the 0.009 mmol L^−1^ step. The q_p_ values are an average of the duplicate experiments. The 0.025 mmol L^−1^ DO step lasted approximately 80 h and the DO was not restored to non‐limiting levels

The O_2_ consumption, CO_2_ production, and penicillin production rates decreased during all long‐term DO step down experiments, and these rates have restored to their original values after the DO was restored to a non‐limiting level. Lower DO values (<0.025 mmol L^−1^) resulted in a more progressive decrease of these rates (Figure 3, Figure 4, and Supplementary Material A Figure A1).

The influence of low DO on the penicillin pathway in *P. chrysogenum* was analyzed by quantifying the concentrations of the pathway intermediates and by‐products. The results of the metabolite concentration measurements of the reference experiment and the 0.025, 0.013 and 0.009 mmol L^−1^ step experiments are presented in the Supplementary Material B, Figure B1‐B6. The results clearly indicated that the flux through IPNS decreased at low DO, represented by the decrease in IPN and increase in both intra‐ and extracellular ACV and bisACV levels. Regarding the precursor amino acids, the AAA concentration increased during the 0.009 mmol L^−1^ step experiment, the valine concentration showed a quick initial increase and a gradual decrease trend, and the concentration of cysteine did not change. A more detailed description of the pathway metabolite levels is presented in Supplementary Material B.

### 3.3 Oscillation experiments

With the aim to describe how the *P. chrysogenum* cells respond to an imperfectly mixed large‐scale bioreactor environment where oxygen rich and poor zones exist, two different oscillation experiments were conducted where experiment II imposed more severe oxygen starvation than that in experiment I. Understanding the metabolic responses as quantified by the pathway metabolite levels and extracellular C_x_, C_s_ and C_p_ values was the first step in pinpointing possible metabolic bottlenecks that could further lead to an improved penicillin production process. The results of these experiments were also used to validate our model.

**FIGURE 5:**
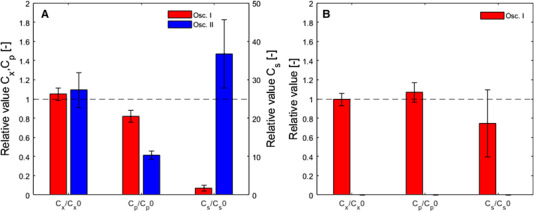
Relative C_x_, C_p_, and C_s_ values collected during the two oscillation experiments as compared to the steady state condition. (A) Values measured at the end of the oscillation phase and (B) after the oscillation phase when the DO was restored to non‐limiting values. The DO was only restored in the oscillation experiment I

Both of the oscillation experiments indicated a slight increase in C_x_ during the oscillation period, which returned to the original value after the DO was restored to a non‐limiting steady value (Figure 5, Figure B2 in Supplementary Material B).

When analyzing the penicillin production rates, we found that the periodically recurring DO values of zero mmol L^−1^ in both oscillation experiments resulted in a decreased q_p_, which was inferred from the decreased extracellular penicillin concentration (Figure 5) and from the calculated biomass specific penicillin production rates (Supplementary Material A, Figure A3). The difference in q_p_ between the two oscillation experiments showed that the longer oxygen starvation exposure time (experiment II) resulted in a lower q_p_ even though the cycle mean was the same in both experiments. After the DO was restored to its original values, the q_p_ increased to that observed under the initial steady state condition in experiment I.

The metabolite level measurements are presented and described in Supplementary Material B, Figure B7‐B9. During both oscillation experiments the ACV concentration remained steady and no bisACV was detected. The extracellular and intracellular IPN concentrations decreased during the oscillation phase. Both the penicillin concentration in the broth and the extracellular IPN level restored when the DO was increased above 0.136 mmol L^−1^. While the cysteine concentrations remained steady, the AAA concentrations slightly decreased and the valine concentration showed a quick increase and a slow decrease to the original value during the oscillation phase.

### 3.4 Modeling the effect of O_2_

In order to obtain the biomass specific rates of respiration, sugar uptake, growth, and penicillin production, and better understand the metabolite concentration profiles obtained under different DO levels, the metabolism of the cells was modeled. Initially, a black box model was created to link the DO level to respiration rates and then linked to a penicillin pathway kinetic model [44] to serve as a basis for describing the central metabolism. The kinetic model resulted in a detailed prediction for the penicillin production rate, which considered several enzymatic conversion steps. The two models were linked together via the predictions of C_x_, C_p_ and C_s_.

With the black box model, we aimed to link the primary and secondary metabolism of the cells to low DO conditions. The model predicted the growth, sugar uptake, oxygen uptake, carbon dioxide emission, and penicillin production rate as a function of DO. The oxygen‐related model parameters were then estimated based on the experimental data.

In the model, the distribution of oxygen and carbon between growth, maintenance, penicillin production, and by‐product formation were evaluated according to Herbert‐Pirts law and hyperbolic kinetics was used for both sugar and oxygen consumption. Both the effects of low oxygen concentration on the central metabolism, as expressed by limitation of the respiration rate, and the influence on penicillin production rate via IPNS were included. Additionally, to predict the penicillin productivity of a reactor, the effects on the glucose and biomass concentrations were also taken into account, since the penicillin gene cluster is inhibited by glucose [24], and the glucose requirements of penicillin production influence cell growth rates. A saturation constant of the cellular oxygen uptake rate (K_o_^qo^) was introduced into the hyperbolic kinetic model, which represented the DO value at which the oxygen uptake rate decreased to half of its maximum value; thus, determining the change in the biomass specific oxygen uptake rate at different DO values. in this model, K_o_^qo^ was estimated from the experimental data, including both the short DO perturbation (oxygen uptake rates) and step experiments (biomass, residual sugar and penicillin concentration measurements).

The activity of the second enzyme in the penicillin pathway, IPNS, is influenced directly by oxygen concentration because this step consumes molecular oxygen. Bainbridge et al. reported a linear relationship between IPNS activity and the O_2_ concentration within the range of 0.07 to 0.18 mmol L^−1^ [23], which was used in modeling studies [21, 22, 45]. However, at lower O_2_ concentrations (<0.07 mmol L^−1^), the effect of DO on the rate of the IPNS enzyme has not yet been investigated. In our study, we reported that the oxygen dependency of IPNS enzyme followed hyperbolic saturation kinetics at DO levels in the 0.009‐0.025 mmol L^−1^ range.

The penicillin pathway model was based on Deshmukh et al. [44], to which the influence of varying external sugar and oxygen concentrations were incorporated. In this model, the IPNS enzyme followed hyperbolic saturation kinetics with respect to oxygen concentration, with an affinity parameter K_o_^IPNS^ that was estimated based on our data, and the gene cluster that was inhibited by high glucose concentrations. To predict the sugar and biomass concentrations, a black box approach was used, thereby coupling the two models. K_o_^IPNS^ was estimated from the measured penicillin concentration patterns during the DO step experiments. The model equations and estimated values of the parameters are presented in the Appendix.

### 3.5 Simulation results

#### 3.5.1 Simulation of the step experiments

**FIGURE 6:**
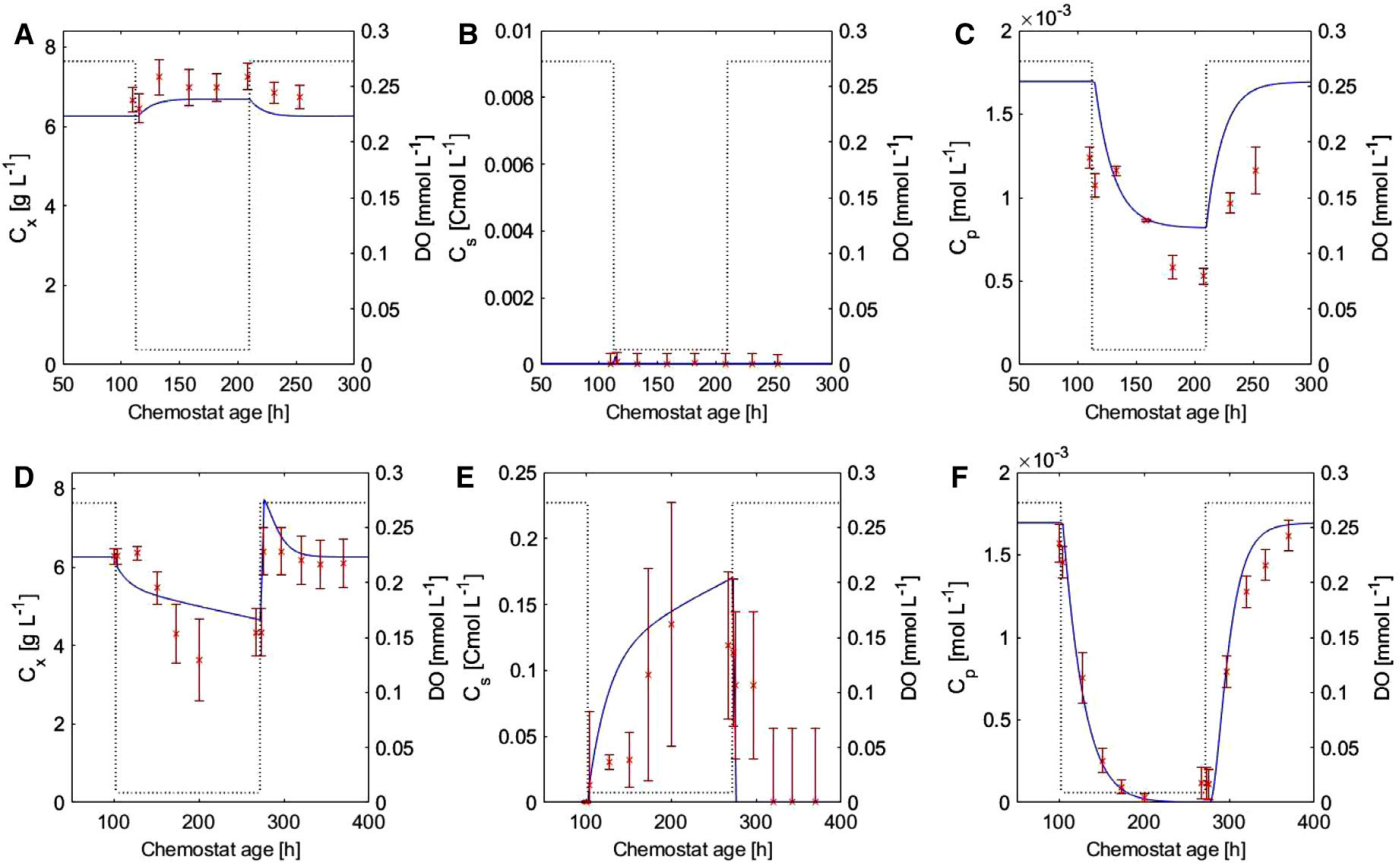
The C_x_, C_s_, and C_p_ profiles at 0.013 mmol L^−1^ (A–C) and 0.009 mmol L^−1^ DO (D–F). The solid line represents the simulation, the crosses represent the measurements, and the dotted line indicates the DO applied in the model. The measured data points are calculated as averages between the duplicate step experiments. The error bars represent the standard deviation of multiple measurements and the duplicate reactor runs

With the developed model, chemostat experiments were simulated during the DO step experiments. The obtained simulation results and measured values of C_x_, C_p_, and C_s_ are presented in Figure 6. The observed opposing trends in C_s_ and C_x_ at growth limiting and non‐growth limiting DO were reproduced by the model. The model predicted well the penicillin concentration and also the penicillin pathway metabolite levels. In particular, the IPN was recused and ACV and bisACV accumulated in low DO conditions (Supplementary material B, Figure B10). The contribution of catabolite repression and reduced IPNS activity to the reduction of the penicillin production rate is shown in Figure B15, Supplementary material B.

#### 3.5.2 Simulation of the oscillation experiments

**FIGURE 7:**
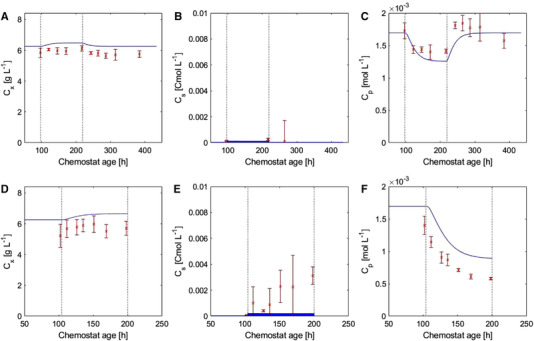
C_x_, C_s_, and C_p_ during oscillation experiments I (A–C) and II (D–F). The red crosses indicate the measurements, the blue line represents the model simulation, and the dotted lines indicate the start and end of the DO oscillation phase

The simulations of C_x_, C_s_, and C_p_ during the oscillation experiments were consistent with the experimental results (Figure 7). According to the modeled predictions, the increase in C_x_ was relatively small and fell within the error margin of the measurements and therefore, was not clearly detectable experimentally. The small experimentally observed increase in C_s_ during the 100 h time frame of oscillation experiment II was not predicted by the model; however, the model predicted a variation of C_s_ within a cycle. The decrease in C_p_ in the simulations was due to both the direct influence of the low oxygen concentrations on IPNS, and the accumulated extracellular sugar within a cycle that repressed the penicillin gene cluster (Supplementary material B, Figure B15). The simulation of the pathway metabolites are presented in the Supplementary material B, Figure B11. While the IPN accumulation was predicted well within the oscillating DO conditions, the ACV and bisACV accumulated in the model predictions, which was in contrast to the experimental measurements.

## 4 DISCUSSION

### 4.1 Metabolic response to low DO: Short‐ and long‐term DO perturbation experiments

The short and long DO step experiments showed that with decreasing DO, penicillin production was limited, possibly through the oxygen limitation of IPNS. The penicillin concentrations showed the most significant reduction in the penicillin production rate at the lowest oxygen concentration. The expected mechanism behind the decrease in q_p_ was the decreased rate of IPNS enzyme due to oxygen limitation [23], which was apparent from the measured metabolite levels, where the IPNS concentration decreased while the intracellular ACV accumulated, both of which were most affected by the lowest DO level. These observations are in contrast to the measurements of Henriksen et al., who reported increased IPN concentrations under low DO concentrations of 0.019–0.344 mmol L^−1^ [15]; however, previous findings of ACV accumulation in the intra‐ and extracellular space is consistent our observations [15, 21]. Our results suggested that at DO concentrations between 0.013 and 0.025 mmol L^−1^, the IPN levels decrease to values that approach or fall below the Km values of the IAT and IAH enzymes, which reduce penicillin production.

The reduced q_p_ at low oxygen concentrations (0.013–0.025 mmol L^−1^) resulted in an increase of the biomass yield. This result can be explained by the reduced penicillin production rate under low oxygen concentrations, which increased sugar availability for cell growth. According to the model predictions, the biomass‐specific sugar uptake decreased because of the reduction in the biomass specific penicillin production rate. However, with the elevated biomass concentration, the overall sugar uptake increased, resulting in a decreased residual sugar concentration that corresponded to a reduced specific glucose uptake rate, which was described by saturation kinetics. Similarly, reduced penicillin production rates under sugar limitation could have reduced the biomass specific oxygen requirements, as penicillin production requires O_2_. The experimentally observed gradual decrease of the penicillin pathway flux might have been caused by changes in the enzyme levels of the penicillin pathway over time because enzyme expression is a slower process than enzyme limitation.

Further decrease of the DO below 0.013 mmol L^−1^ affected the primary metabolism of the cells as well. This was observed as a significant decrease of the respiration rate (q_O2_ and q_CO2_), indicating that oxidative phosphorylation was limited by oxygen availability. Between a DO of 0.013 and 0.009 mmol L^−1^, a shift from glucose‐ to oxygen‐limited growth occurred and resulted in an increased residual glucose concentration at 0.009 mmol L^−1^ DO. Consequently, the growth rate declined to a value below the dilution rate and resulted in wash out of the cells, as indicated by the decreased biomass concentration.

At a DO of 0.009 mmol L^−1^, the excretion of by‐products increased compared with the excretion at non‐limiting DO values. At 0.009 mmol L^−1^ DO, the biomass concentration declined, suggesting the observed organic carbon excretion might have been a result of cell death and autolysis, a phenomenon that has been reported at low DO concentrations in the filamentous fungi genus, *Aspergillus awamori* [46]. Additionally, the cellular metabolism might have shifted towards by‐product formation as a result of overflow metabolism. It has been reported that at low oxygen concentrations, the excretion of polysaccharides increases in higher‐mushrooms [47] and similarly, that the total carbohydrate content in the culture filtrate of *Penicillium chrysogenum* increased at low DO [16].

The increased residual glucose concentration at growth limiting DO levels could have resulted in the repression of penicillin producing enzymes. In the 0.009 mmol L^−1^ DO step experiment, the effect of sugar repression on the penicillin gene cluster and the reduced IPNS activity could have played a significant role in reducing q_p_. The IPN levels decreased to values close to the Km of the IAT enzyme for IPN; therefore, a decrease in the direct flux towards Penicillin‐G was expected. Additionally, the IPN concentration was reduced to a value 200 times below the Km for IAH, suggesting that almost no 6APA formation took place, further reducing flux towards penicillin production.

In contrast to previous results [18], the observed respiration rates during the short DO perturbation experiments in this study suggested that the respiration rate followed the saturation kinetics with the dissolved oxygen concentration, and by applying this assumption in our simulations, the predicted q_O2_ and q_CO2_ results resulted in a good fit with the experimental data. The observed q_O2_ reduction with decreased DO in the long‐term step experiments were similar to those observed in the short perturbation experiments. Additionally, these results were similar to those reported by Vardar and Lilly [14], as indicated in the Supplementary Material A, Figure A4. In our study, the influence of low DO values on the q_O2_ was more pronounced compared to those observed by Henriksen et al. [15], since the lowest DO tested in their study was 0.019 mmol L^−1^, which did not show a reduction in q_O2_ [15]. The differences between these and our results may be related to the differences in *P. chrysogenum* fungal strains used in the two studies.

The developed model described the experimentally obtained C_x_, C_s_ and C_p_ values well during the step experiments with a saturation constant for respiration K_o_^qo^ of 0.014 mmol L^−1^, which was estimated from the data from the short‐term DO perturbation experiments. The obtained oxygen saturation constant was slightly higher compared to the reported value of 0.003 mmol L^−1^ for *Aspergillus niger* [48]. However, this is still in the range of fungal cells as summarized by Cui et al. [49].

When the DO was restored after the low DO steps, the penicillin, biomass and residual sugar concentrations, and the oxygen uptake and carbon dioxide emission rates were restored to the original values that corresponded to the initial steady state conditions in all step experiments. The recovery of respiration in *P. chrysogenum* after exposure to limiting oxygen concentrations was previously reported [16, 17, 18]. Our observation of the fast restoration of q_p_ after oxygen limitation recovery is consistent with previous observations, where instantaneous recovery was observed after the DO was increased to 0.08 mmol L^−1^ from a 0.019 mmol L^−1^ [15]. However, our results were not consistent with those of Vardar and Lilly, who reported an irreversible and complete cessation of q_p_ at DO values below 0.027 mmol L^−1^ [14]. Similarly, the metabolite levels confirmed that the reduced penicillin production was reversible in our experiments as after a 100 h period of exposure to low DO conditions, which restored the metabolite levels to their initial values.

### 4.2 Oscillation experiments

Both oscillation experiments in which the DO varied within 2 min cycles between limiting and non‐limiting values resulted in a more severe reduction in q_p_ compared to a steady DO of the average of the cycles. At this average DO (0.06 ± 0.002 mmol L^−1^) under steady conditions, neither q_p_ nor q_o_ was expected to be notably influenced according to both model predictions and experimental data, both of which indicated that during the DO step experiment at 0.054 mmol L^−1^ the penicillin production was not affected (Supplementary Material A, Figure A1). This observation was similar to that of Vardar and Lilly [14], where the DO was cycled around 0.08 mmol L^−1^, which decreased q_p_ to lower values than those observed at a steady DO that represented the average of the cycles. Thus, an oscillating DO level might be less efficient than a steady average DO condition with respect to penicillin production. This could be due to the periodic limiting oxygen levels in which the average IPN synthesis rate slows down and affects the overall pathway flux towards penicillin. According to the simulations, both the reduced IPNS rate and the fluctuating sugar concentration contributed to the decrease in q_p_ during the oscillation experiments. The simulation results showed that the q_p_ observed in oscillation experiment I would be reached at a steady DO of approximately 0.03 mmol L^−1^, while in the oscillation experiment II, this result would require a steady DO of 0.014 mmol L^−1^. The decline of q_p_ took place within the first 24 hours of DO oscillations, where after it stabilized, which also matched the observations of Vardar and Lilly [14]. During the experiments, the biomass concentration showed a slight increase. This trend is similar to the non‐growth limiting step experiments and is reasoned by the reduced penicillin production rates.

Similar to the long‐term step experiments, the C_x_, C_s_, C_p_, and q_O2_ were restored after the DO oscillations were terminated and the DO returned to non‐limiting values. These observations match previously reported results, where q_p_ recovered after a day of repeated cycles of 0.082 ± 0.019 mmol L^−1^ [14]. Additionally, decreases in q_O2_ were reversible after the cells were exposed to complete oxygen starvation for 1–2 min [18]. However, cycles that included long O_2_ starvation (10 min) significantly affected the oxygen uptake rate in an irreversible manner [18], which was consent with our oscillation experiments that showed complete oxygen starvation (zero mmol L^−1^ DO) was only achieved for short time periods of less than a minute.

The effect of oscillations on penicillin pathway metabolites differed from the step experiments. The main difference was that during the oscillation experiments, no accumulation of intracellular ACV was observed. In our model simulations, the IPNS flux decreased below the pathway flux during the oscillations and therefore, ACV accumulation was expected. However, the complex interactions between the primary and secondary metabolism could have affected the metabolite levels and explain the obtained q_p_. The dependence of q_p_ on the central metabolism can be affected by the energy, precursors, and cofactors required for the penicillin production pathway [50]. Therefore, possible reasons for the lack of ACV accumulation are insufficient amino acid or energy supply for the conversion. It has been reported that amino acid synthesis, especially cysteine biosynthesis, can limit the pathway flux [51]; however, our measurements indicated steady cysteine and valine concentrations. In contrast to the 0.009 mmol L^−1^ DO step experiment, the intracellular AAA concentration decreased only slightly during oscillation experiment I. Therefore, the possibility that the central metabolism limited the AAA biosynthesis and ACVS rate cannot be excluded. The lack of ACV accumulation could correlate to the energy supply for the enzymatic conversion step catalyzed by ACVS; the ACVS enzyme requires ATP [52] and the ATP supply could be reduced as a consequence of the decrease in average glycolytic and TCA flux.

## 5 CONCLUDING REMARKS

This study provides detailed insight into the metabolic response of *P. chrysogenum* to periodic oxygen limitations, and can be used to model the fermentation process under heterogeneous oxygen conditions as observed in large‐scale reactors. Fermentations were monitored during step‐wise changes in DO and during oscillating DO conditions. The metabolite levels of the penicillin pathway were also evaluated. The experiments showed that at a DO of 0.009 mmol L^−1^, the central metabolism was limited, while between 0.009 and 0.25 mmol L^−1^, only penicillin production was reduced. During oscillating DO conditions that are experienced by *P. chrysogenum* cells under large‐scale reactor conditions, the penicillin production rate decreased below the values expected in stable DO levels equal to the average cycling DO. A decrease in penicillin production, sugar and oxygen uptake, and growth rates due to low DO were reversible when the DO levels were restored to non‐limiting values (>0.136 mmol L^−1^). In order to predict the cellular metabolic response to varying DO levels, the experimental results were modeled with a coupled black box model and detailed kinetic model of the penicillin pathway. During a quasi‐steady state at low DO, the reduced penicillin production rate and altered metabolite levels were well described by the decreased IPNS rate and the inhibition effect of the accumulated extracellular sugar on the penicillin gene cluster. During oscillating DO conditions, the observed lack of ACV accumulation could be explained by the limited availability of precursors or energy supply from the central metabolism. The model predicted the observed respiration, growth, sugar uptake, and penicillin production rates during step‐wise DO changes, as well as during scale‐down conditions. Thus, the developed model can be applied to predict cellular behavior in large‐scale bioreactors where insufficient mixing takes place and the substrate levels show fluctuations in space and time.

## CONFLICTS OF INTEREST

This project is sponsored by Centrient Pharmaceuticals, a producer of lactam antibiotics, and DSM a global company active in Nutrition, Health & Sustainable Living.

## Supporting information

Supporting Information

Supporting Information
