# Supplementary material for: Influence of oxygen concentration on the metabolism of Penicillium chrysogenum
Source: Eng Life Sci. 2022 Apr 7;23(1):e2100139. doi: 10.1002/elsc.202100139 (PMC9815084; doi:10.1002/elsc.202100139)
Supplement: Supplementary file 1 — Supporting Information [file ELSC-23-e2100139-s001.pdf]

Supplementary Material A : C_x, C_s, C_p, q_p, q_{O2} and morphology

Biomass, residual sugar and penicillin concentrations

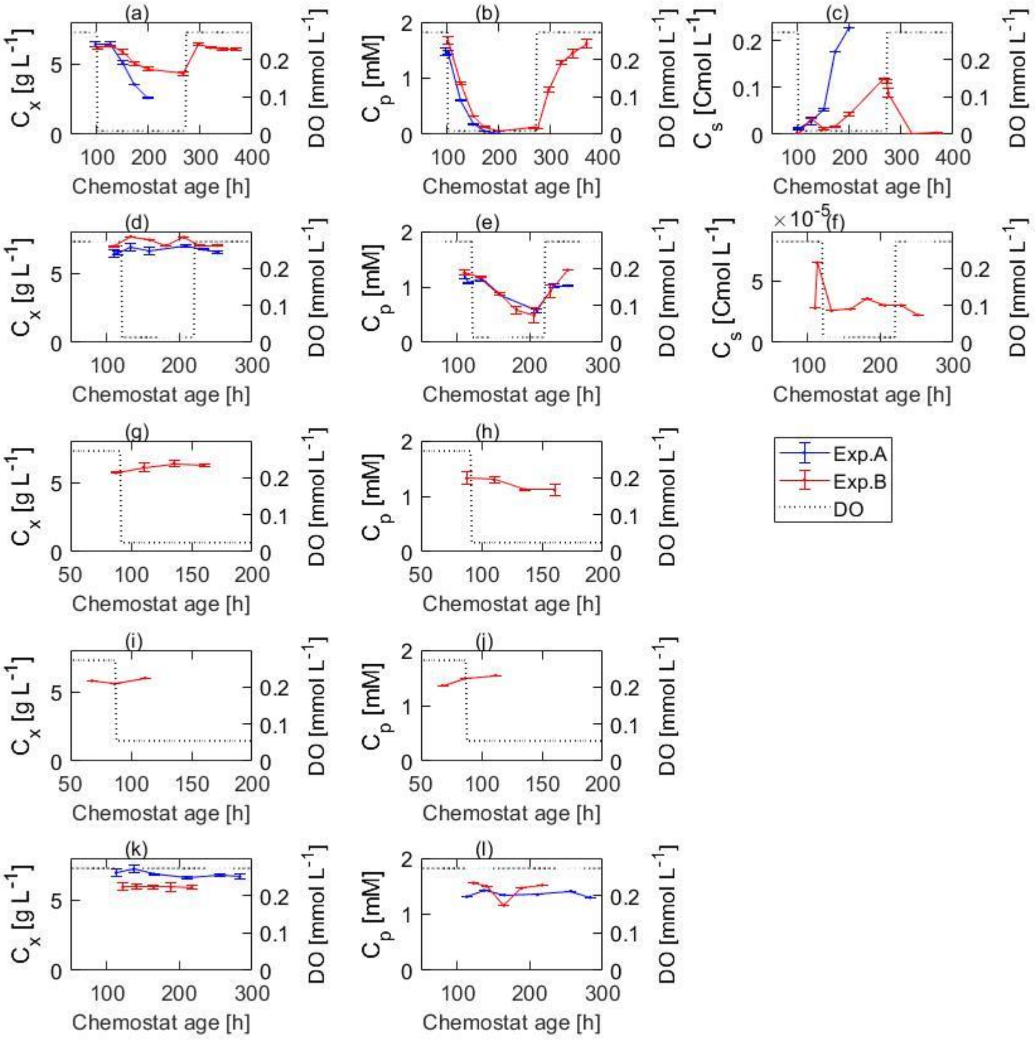

Figure A1. Experimental C_x, C_p and C_s values during the reference and DO step experiments. a)- c): 0.009 mmol L^{-1} DO step experiment; d)- f): 0.013 mmol L^{-1} DO step experiment; g)- h): 0.025 mmol L^{-1} DO step experiment; i)- j) : 0.054 mmol L^{-1} DO step experiment; k)-l): Reference experiment at steady, non-limiting (>0.136 mmol L^{-1}) dissolved oxygen concentrations. Time zero indicates the start of the chemostat phase. The dotted lines represent the aimed DO level, while the red and blue lines are the measurements from the duplicate fermentations.

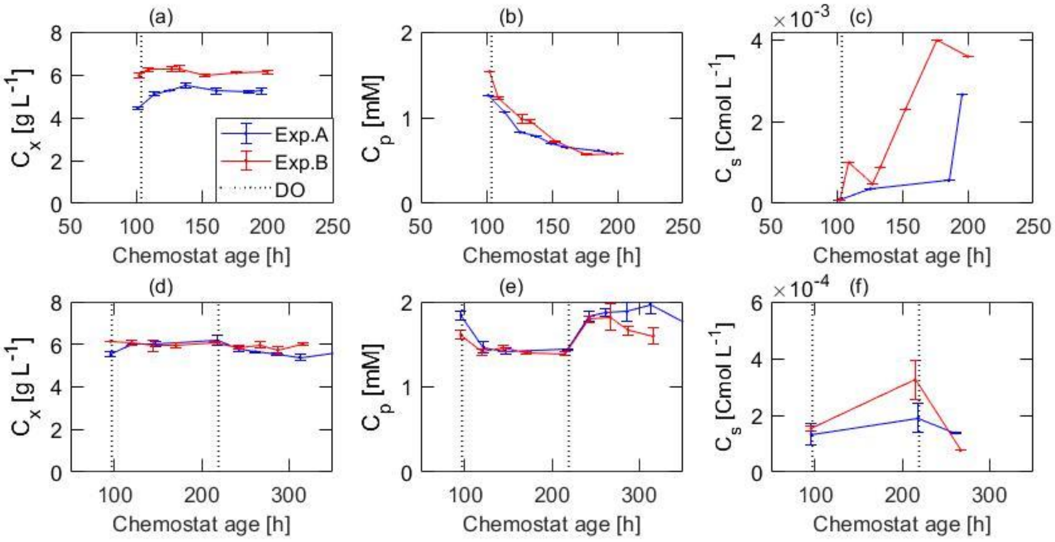

Figure A2. Experimental C_x, C_p and C_s values during the oscillation experiments. a)- c): oscillation experiment I; d)-f): oscillation experiment II. Time zero indicates the start of the chemostat phase. The dotted lines represent the start and end of the oscillation phase, while the red and blue lines are the measurements from the duplicate fermentations.

Relative q_p during the oscillation experiments

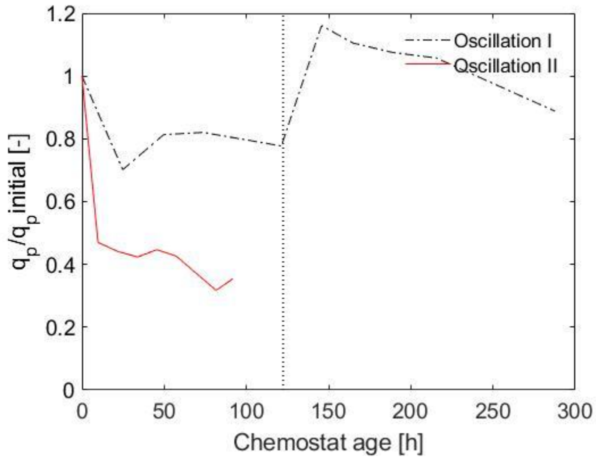

Figure A3. Calculated relative penicillin production rate during the oscillation experiments. Relative values indicate that the q_p values were normalized to the values achieved during the initial steady state conditions at non-limiting DO levels (>0.136 mmol L^{-1}). Time point zero represents the start of the oscillation phase. The vertical dotted line represent end of the oscillation phase of the oscillation experiment I, where the DO was restored to non-limiting values. The q_p values were averaged for the duplicate experiments.

Oxygen uptake rate

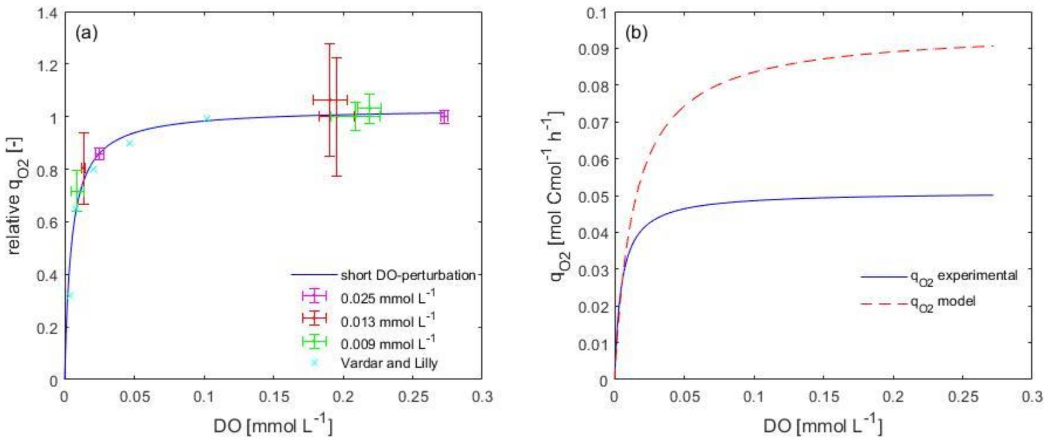

Figure A4. a) Relative biomass specific oxygen uptake rate during the step experiments and the short DO-perturbation experiment, normalized to the values obtained during the initial, steady-state conditions under non-limiting DO levels. The curve represents hyperbolic saturation kinetics fitted to the experimentally obtained data. Additionally, reported values from Vardar and Lilly are also shown. The markers at DO > 0.19 mmol L^{-1} during the step experiments show the relative respiration rates during the steady state precedent to the steps (value of 1 by definition) and after the step when the DO was restored to the original, non-limiting value. b) hyperbolic saturation kinetics describing q_{O2}, fitted on the experimentally obtained data (solid blue line) and predicted q_{O2} values in O_2 limited cultures (dashed red line).

Morphology

Our daily microscopic analysis during the step-down experiment revealed morphological changes after the step-down in DO to 0.009 mmol L^{-1}, while at higher DO concentrations, no changes were observed. Before the step-down to 0.009 mmol L^{-1}, the cells were freely dispersed, however, after the step-down, big clumps of cells were present in the cell broth (Figure 4). These macroscopic flocs in the broth visibly altered the broth texture, and the biofilm formation was enhanced. These effects were reversed when the DO was restored to non-limiting values. These observations suggest that the morphology of the cells is influenced by the DO level. Since the cells might face lower DO values in the middle of the flocs compared to the cells situated on the outer-layers due to diffusion-limitations, the values of the saturation constants (K_o^{IPNS} and K_o^{qo}) may represent the average DO levels that the cells are exposed to

within a floc. These values therefore may not describe the properties of the individual cells, rather the flocs, and thus the saturation constant of individual cells might be slightly lower.

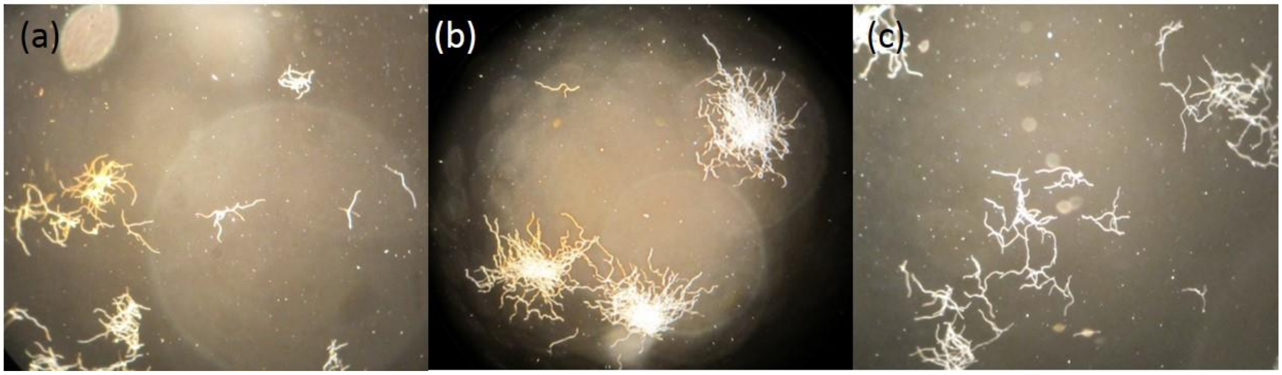

Figure A5. Pictures taken under the microscope during the 0.009 mmol L^{-1} DO step experiment, a) taken before the step down, b) at 100 h after the 0.009 mmol L^{-1} step down, c) picture taken after the DO was restored.
