# Supplementary material for: Influence of oxygen concentration on the metabolism of Penicillium chrysogenum
Source: Eng Life Sci. 2022 Apr 7;23(1):e2100139. doi: 10.1002/elsc.202100139 (PMC9815084; doi:10.1002/elsc.202100139)
Supplement: Supplementary file 2 — Supporting Information [file ELSC-23-e2100139-s002.pdf]

Supplementary Material B: Metabolite levels, DO curves and simulations

Metabolite levels involved in the penicillin pathway during the step experiments

The measured intracellular metabolite concentrations clearly indicate that the flux through IPNS is decreasing at low DO, as the ACV levels increase and the IPN levels decrease as a result of a step in the DO (Figure B2, B3, B4). Also the extracellular concentrations of these metabolites were affected after the step down in DO, as can be seen from the measurements at 0.025 and 0.009 mmol L^{-1} DO (Figure B5 and B6, respectively). Next to intracellular ACV accumulation, bisACV formation also took place during the step experiments. Since ACV is a reactive compound due to its thiol moiety, it might form disulfide bonds with other ACV molecules by oxidation, forming an ACV-dimer, bisACV. ACV might also react with other thiol compounds, which were not quantified. During sampling for intracellular metabolite quantification, ACV was protected from oxidation by the addition of maleimide, a derivatizing agent [1], while no treatment was applied to the extracellular samples. Therefore, the extracellular bisACV might be due to either ACV or bisACV excretion [2], thus representing the sum of the extracellular concentrations of these compounds, and this sum might be slightly underestimated due to the formation of unquantified thiol compounds [2]. During the 0.013 mmol L^{-1} step, the intracellular ACV concentration increased gradually. Similarly, the intracellular bisACV concentration increased in a similar manner as the intracellular ACV concentration did (Figure B3). In contrast, at 0.009 mmol L^{-1} DO, the intracellular ACV increase was transient, and a peak of ACV was visible at 25 h after the DO step-down (Figure B4). At the 0.009 mmol L^{-1} step, the peak of ACV 25 h after the step-down coincided with a peak in the concentration of intracellular bisACV. At this time-point, also bisACV in the extracellular medium was observed (Figure B6). The extracellular bisACV detected during the 0.009 mmol L^{-1} DO step experiment accounted for more than 50% of the total measured ACV and bisACV.

The extracellular concentrations of metabolites further down the pathway from IPN (such as 6APA and penicillin) followed similar trends as the IPN concentration and decreased during the steps (Figure B5 and B6). Also the intracellular 6APA concentration coincided with the decrease in the IPN concentration at the 0.013 mmol L^{-1} step experiment, while for the other step experiments only the extracellular concentrations showed variation. In parallel to the decrease in extracellular 6APA, also the extracellular concentration of its carboxylation product, 8HPA, decreased (Figure B6).

Although the intracellular Penicillin-G concentrations did not change (Figure B2-B4) the extracellular concentrations declined as a result of the decreased pathway flux (Figure B5 and B6). The extracellular PIO concentration followed the extracellular penicillin concentrations (Figure B6), as PIO is formed from penicillin as a degradation product [3]. Opposing the trend of the extracellular penicillin concentrations, the PAA concentrations showed an increase during the step, both intra-

and extracellularly (Figure B2-B6). Even though the PAA concentrations increased, the oOHPAA concentration declined (Figure B3 and B5). oOHPAA is formed from PAA by hydroxylation, and the phenylacetate-hydroxylase (PAH) enzyme responsible for the conversion requires oxygen [4], explaining the declining oOHPAA concentrations during the DO step down. The extracellular concentration of OPC, which is a cyclic form of α-aminoadipate (AAA) [3], declined during the low DO steps (Figure B6). Regarding the precursor amino acids, the AAA concentration increased markedly during the 0.009 mmol L^{-1} step experiment (Figure B4). The valine concentration showed a quick initial increase and a gradual decreasing trend during the step experiments of 0.013 and 0.009 mmol L^{-1} (Figure B3 and B4). The concentration of cysteine did not change during the DO-step, as indicated by the cysteine measurements at the 0.013 mmol L^{-1} step experiment (Figure B3).

When after the low DO steps the DO was increased back to non-limiting values, the extracellular and intracellular metabolite levels restored to values similar to the ones before the steps (Figure B3, B4 and B6).

Metabolite levels during the oscillation experiments

The extracellular and intracellular IPN concentrations decreased during the oscillation phase. The extra- and intracellular PAA concentrations followed opposite trends compared to the extracellular penicillin concentration and thus increased during the oscillations (Figure B7, B8 and B9). Both the penicillin concentration in the broth and the extracellular IPN level restored when the DO was increased above 0.136 mmol L^{-1} at the end of the oscillation phase in experiment I (Figure B7 and B9). While the cysteine concentrations remained steady (as seen at oscillation experiment II, Figure B8), the AAA concentrations slightly decreased during the oscillation phase (oscillation I, Figure B7) and the valine concentration showed a quick increase and a slow decrease to the original value (as can be seen from the more frequent sampling in oscillation I), similarly to the 0.009 and 0.013 mmol L^{-1} step experiments.

Although the metabolite levels were quantified at approximately 24 h intervals within the time frame of the fermentation, at a few time points several different samples were taken with approximately 10-30 seconds in between (4 intracellular samples were taken around both 98.5 and 213.8 h in oscillation I, and 8 at 200 h oscillation II). This was done to investigate whether metabolite levels changed within a cycle. The results of these measurements (Figure B7 and B8) show that the metabolite levels within a cycle do not deviate significantly from each other. Neither of the metabolite concentrations showed any trend or correlation with the oscillating DO in the

measurements. Similarly, neither of the precursor amino acids showed oscillating behaviours in the second-scale measurement points of the metabolites.

Simulation of the penicillin pathway metabolites during the step experiments

The most important pathway metabolite levels which showed clear dynamics in the measurements (intra and extracellular ACV and bisACV, IPN and extracellular 6APA) are presented in Figure B10, together with the simulated values. The model predicted well the trends of ACV accumulation, excretion, intra and extracellular bisACV formation and accumulation, and intra and extracellular IPN and 6APA decrease as a consequence of low DO. However, the rate of IPN decrease and ACV and bisACV increase at the 0.013 mmol L^{-1} step experiment is faster in the simulations compared to the measurements.

During the 0.009 mmol L^{-1} step experiment, the sugar concentration increase can reason alone the decline in C_p. In contrast, when the influence of C_s is excluded from the penicillin production, the penicillin production is overestimated in our model. The influence of an increased C_s on the enzyme levels at the 0.009 mmol L^{-1} step experiment was apparent from the measured metabolite levels, where the ACV accumulation was transitional: for 25 hours ACV accumulation took place, after which a decrease in the total ACV and bisACV levels in the intra- and extracellular space was observed. This phenomenon was well represented by the model predictions and it might be explained by a reduction of the ACV formation rate. Such a reduction could have been caused by increasing extracellular sugar concentrations which have a repressing effect on the expression of the penicillin gene cluster [5][6], thereby reducing the enzyme levels of the pathway. In case of purely IPNS limitation, an increasing ACV level would have been expected, levelling off at a certain value due to feedback inhibition of the ACVS enzyme [7], such as observed in the 0.013 mmol L^{-1} step experiment. These results seem to confirm that sugar repression of the penicillin production rate occurred during the 0.009 mmol L^{-1} step experiment.

The AAA concentration showed an increase during the 0.009 mmol L^{-1} step experiment, which was in the same range as the decrease of the (extracellular) OPC concentration. The formation of OPC occurs via a yet unknown mechanism, that could either involve enzymatic or chemical conversion [8, 9]. According to our measurements, the OPC concentration followed the same trend as the penicillin concentration, confirming a previously reported hypothesis that the OPC formation rate is linearly related to the penicillin production rate, due to enzymatic conversion of AAA to OPC by one of the pathway enzymes [8]. These findings suggest that the OPC formation is not linearly related to the AAA concentration, as assumed in previous studies [10], but it correlates with the penicillin production rate and the intracellular AAA levels are influenced by these conversions. The sudden

increase in the valine concentration detected after the low DO steps might be explained by a sudden drop in valine uptake both for growth related purposes and for penicillin production, represented as a decline in the ACVS enzyme rate, and later on the accumulated valine was slowly consumed for growth or its production rate was regulated, resulting in a gradual decline in the valine concentration.

Simulation of the penicillin pathway metabolites during the oscillation experiments

Regarding the metabolite levels, the predicted decreasing IPN concentrations matches the measurements. In contrast, the ACV level shows accumulation according to the model while it does not increase according to the measurements. The simulated and measured IPN, ACV and bisACV levels of the oscillation experiment I are presented in Figure B11.

Within a cycle the simulated q_s, q_o and μ changed intensely. In contrast, the q_p did not change significantly in a cycle. Although the IPNS rate changed between 0 and 6000-7000 mmol m^{-3} h^{-1}, the penicillin production rate showed small fluctuations which were less than 5 % of its average value. The rates of the other pathway enzymes also did not change significantly within a cycle, because the IPN concentrations varied within a small range. The ACV and IPN concentrations showed small fluctuations (20-30 mmol m^{-3}) which were smaller than the error of the measurements, and therefore not detectable experimentally. The changes in IPN concentration and IPN and penicillin production rate within a DO oscillation cycle are presented in Figure B13 and B14 for oscillation experiments I and II, respectively.

Intracellular penicillin pathway metabolites during the step experiments

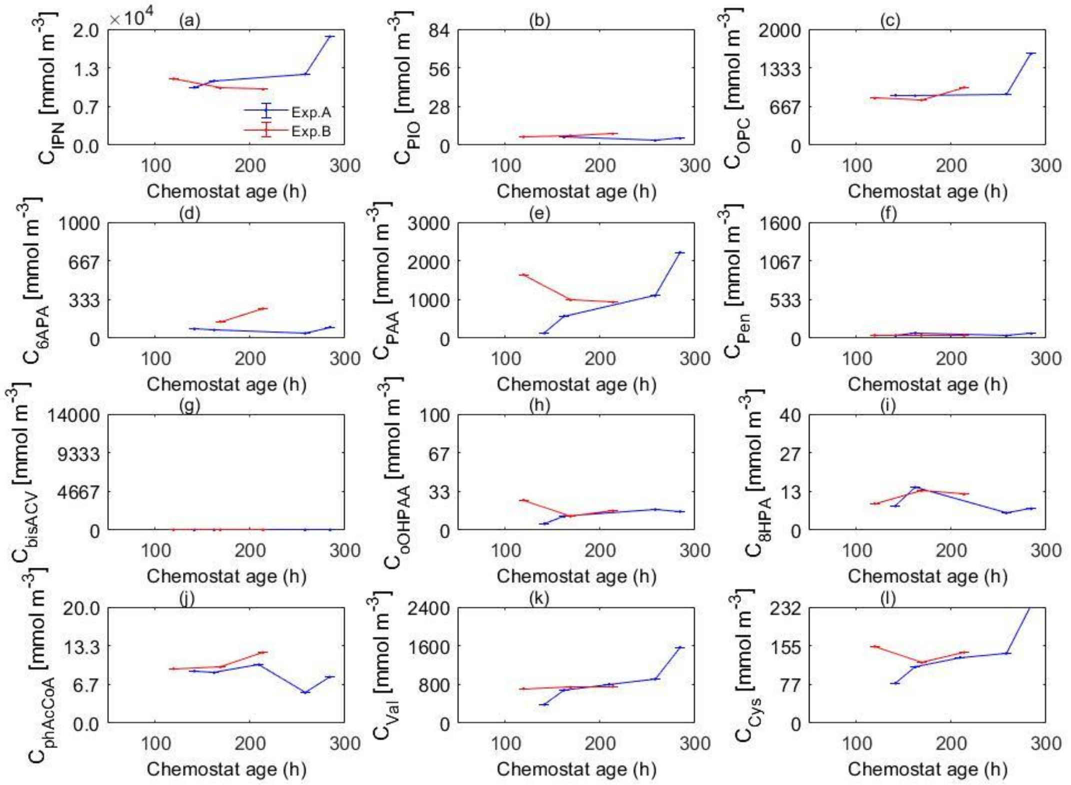

Figure B1. Measured metabolite levels for the reference experiment. Metabolite concentrations were measured in single samples at all measurement points, therefore no error bars are shown. The DO was kept steady at non-limiting values. The red and blue lines show the results of the duplicate fermentations.

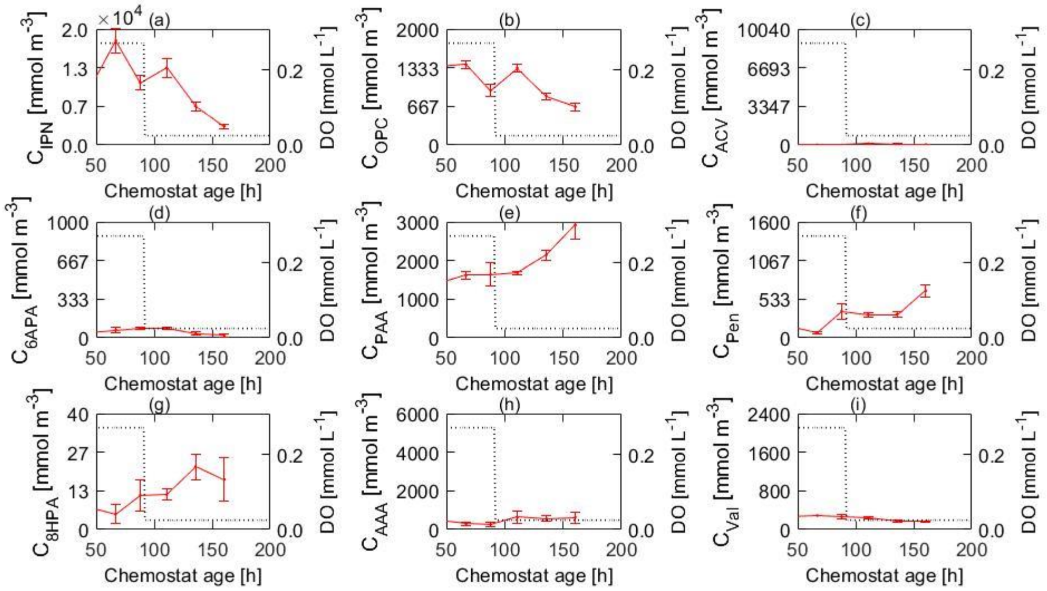

Figure B2. Measured metabolite levels for the 0.025 mmol L^{-1} step experiment. The dotted line represents the simulated DO values, and the red lines show the results of the duplicate fermentations.

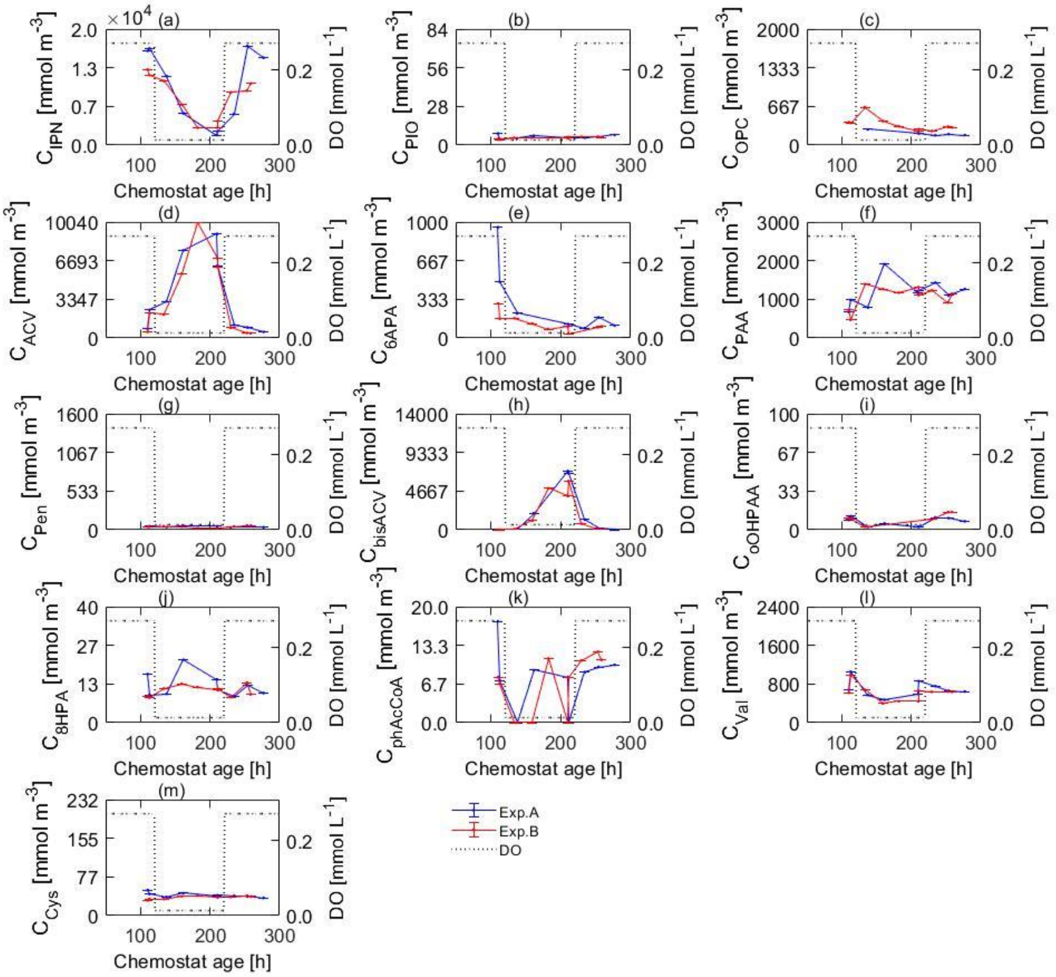

Figure B3. Measured metabolite levels for the 0.013 mmol L^{-1} step experiment. The dotted line represents the simulated DO values, and the red and blue lines show the results of the duplicate fermentations. All metabolite concentrations were measured in single samples at all measurement points, therefore no error bars are shown.

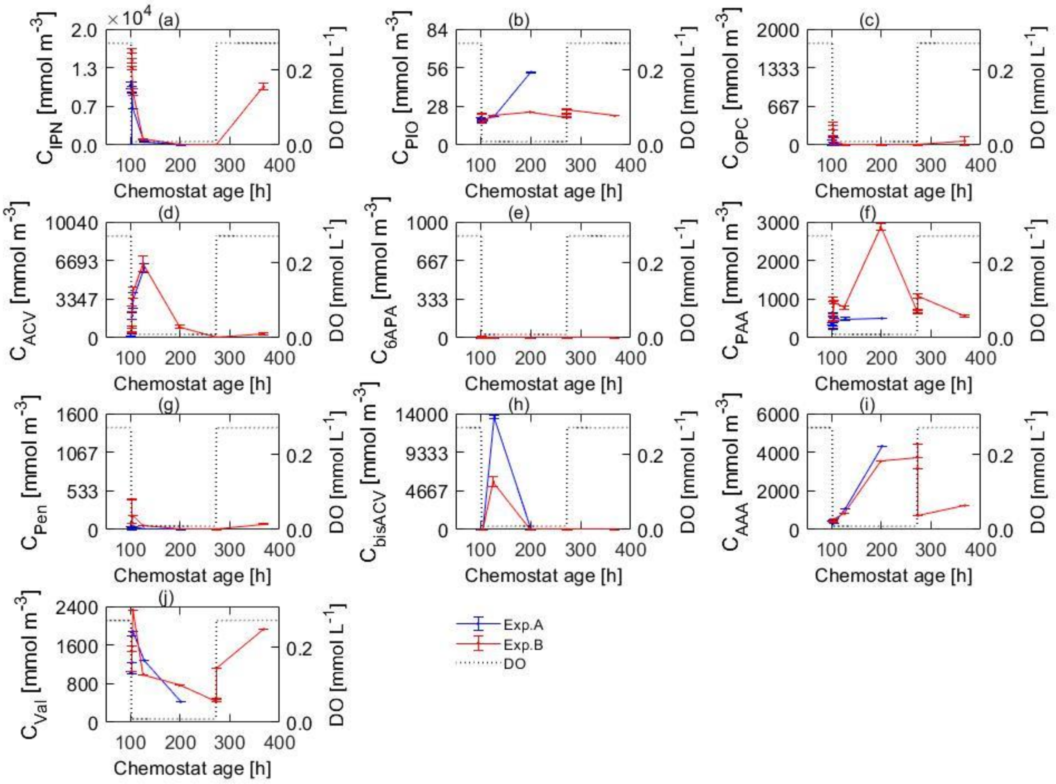

Figure B4. Measured metabolite levels for the 0.009 mmol L^{-1} step experiment. The dotted line represents the simulated DO values, and the red and blue lines show the results of the duplicate fermentations. The AAA and valine concentrations were measured in single samples at all measurement points, therefore no error bars are shown. Right after the step down and step-up, 4 measurement points were taken within less than 2 hours, and plotted in the graphs in order to show short term changes of the metabolites.

Extracellular penicillin pathway metabolites during the step experiments

The extracellular metabolite levels were measured for the 0.009 and 0.025 mmol L^{-1} step experiments. Only those metabolites were measured or could be detected which are shown on the graphs.

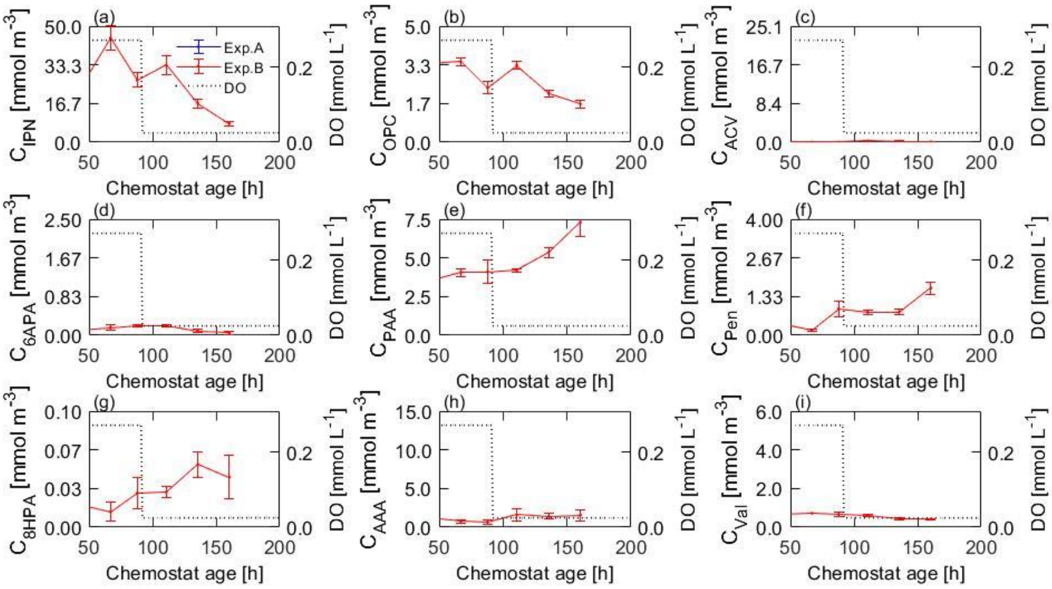

Figure B5. Measured extracellular metabolite levels for the 0.025 mmol L^{-1} step experiment. The dotted line represents the simulated DO.

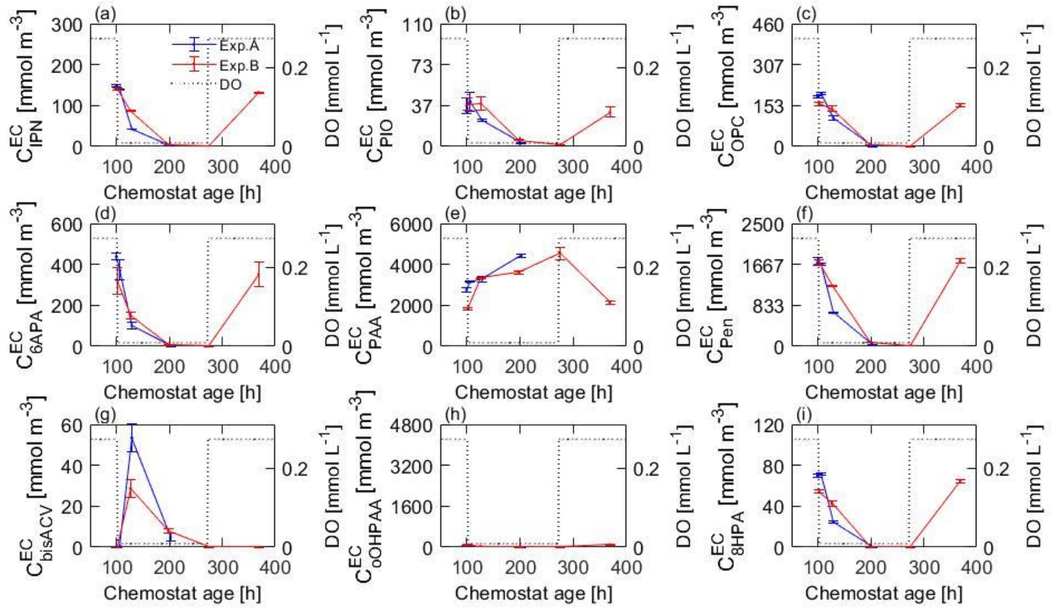

Figure B6. Measured extracellular metabolite levels for the 0.009 mmol L^{-1} step experiment. The dotted line represents the simulated DO values, and the red and blue lines show the results of the duplicate fermentations.

Intracellular penicillin pathway metabolites during the oscillation experiments

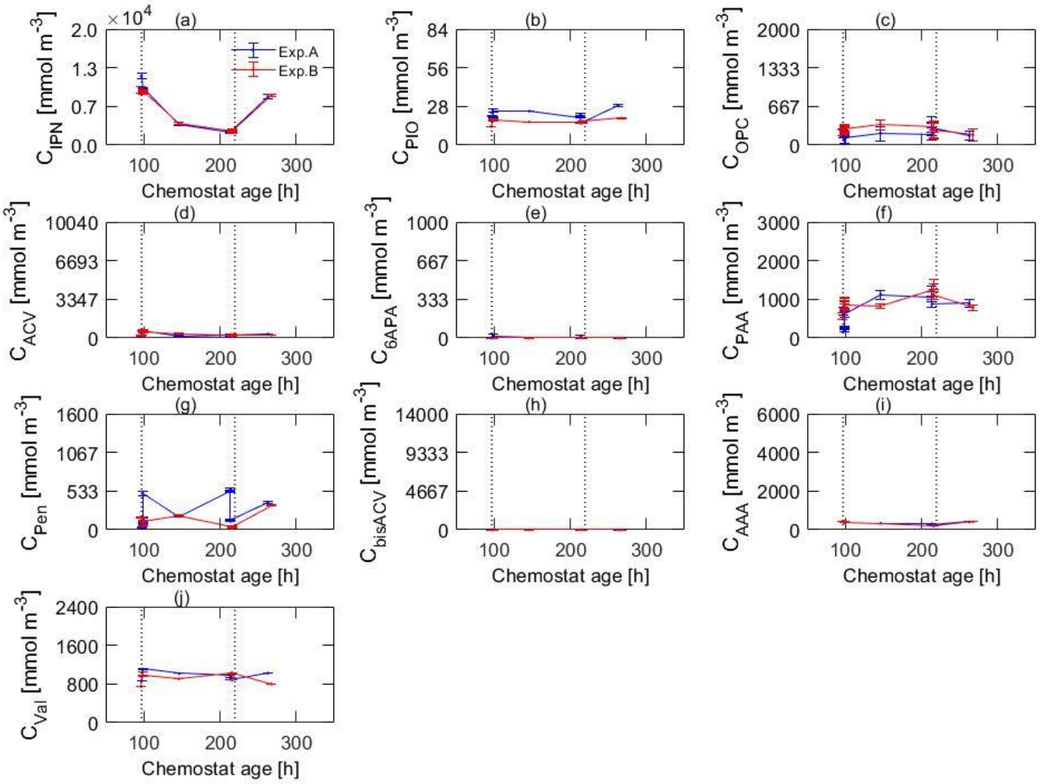

Figure B7. Measured intracellular metabolite levels during the oscillation experiment I. The vertical dotted lines represent the start and the end of the oscillation phase, and the red and blue lines show the results of duplicate fermentations. At the start and end of the oscillation phases, four samples were taken ~30 sec apart from each other, within a DO oscillation cycle, and these points are also plotted. The AAA and valine concentrations were measured in single samples at all measurement points, therefore no error bars are shown.

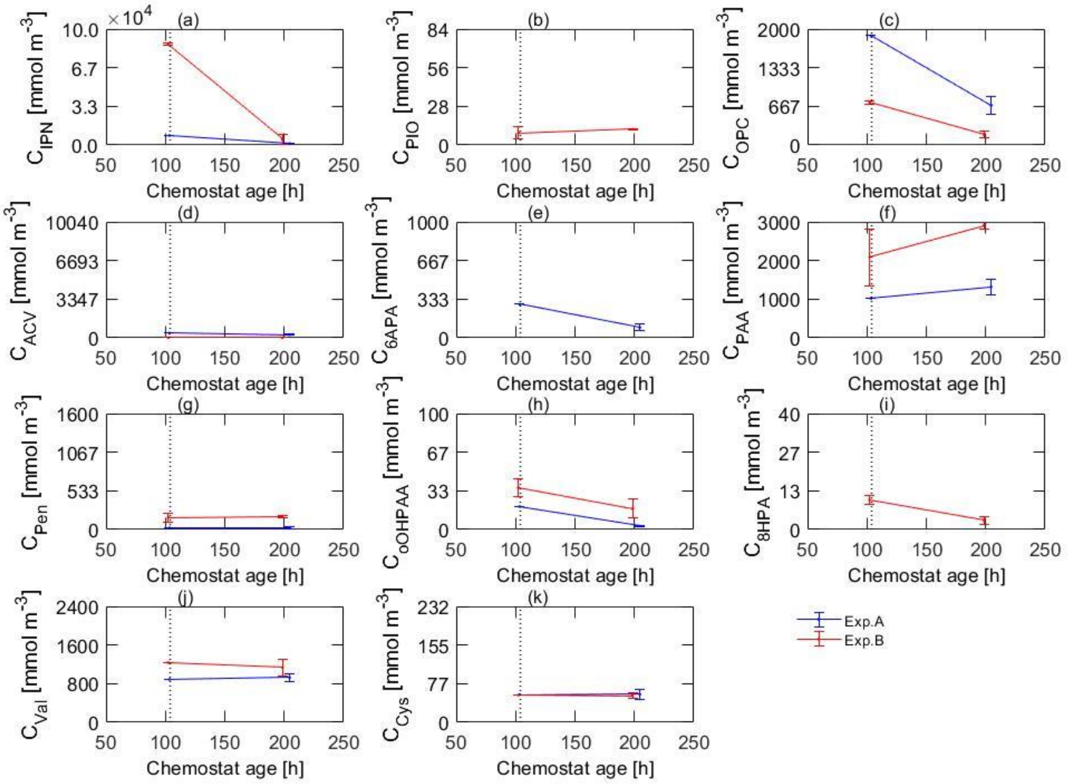

Figure B8. Measured intracellular metabolite levels during the oscillation experiment II. The vertical dotted line represents the start of the oscillation phase and the red and blue lines show the results of duplicate fermentations. The measurements at the steady state with DO levels above 0.19 mmol L^{-1} (at 104 h) are single measurement and therefore no error bars are shown at that time point. The points at the end of the experiment (at 200 h) are averaged metabolite concentrations of several samples taken ~10 s apart from each other during a DO oscillation cycle.

Extracellular penicillin pathway metabolites during the oscillation experiments

The extracellular metabolite levels were measured only for oscillation experiment I. Only those metabolites were measured or could be detected which are shown on the graphs.

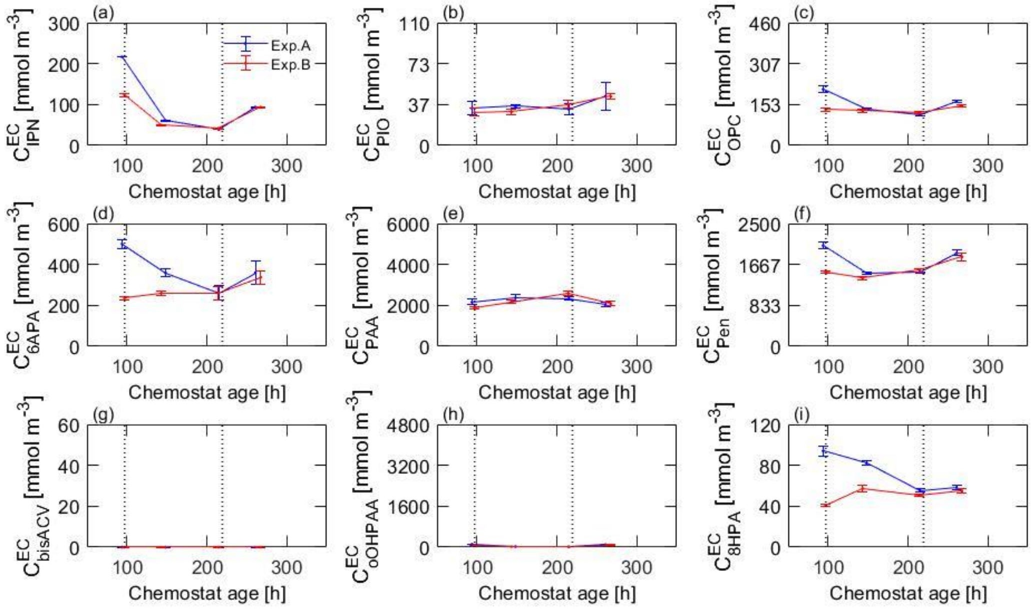

Figure B9. Measured extracellular metabolite levels for oscillation experiment I. The vertical dotted lines represent the start and the end of the oscillation phase, and the red and blue lines show the results of the duplicate fermentations.

Simulation of the step experiments

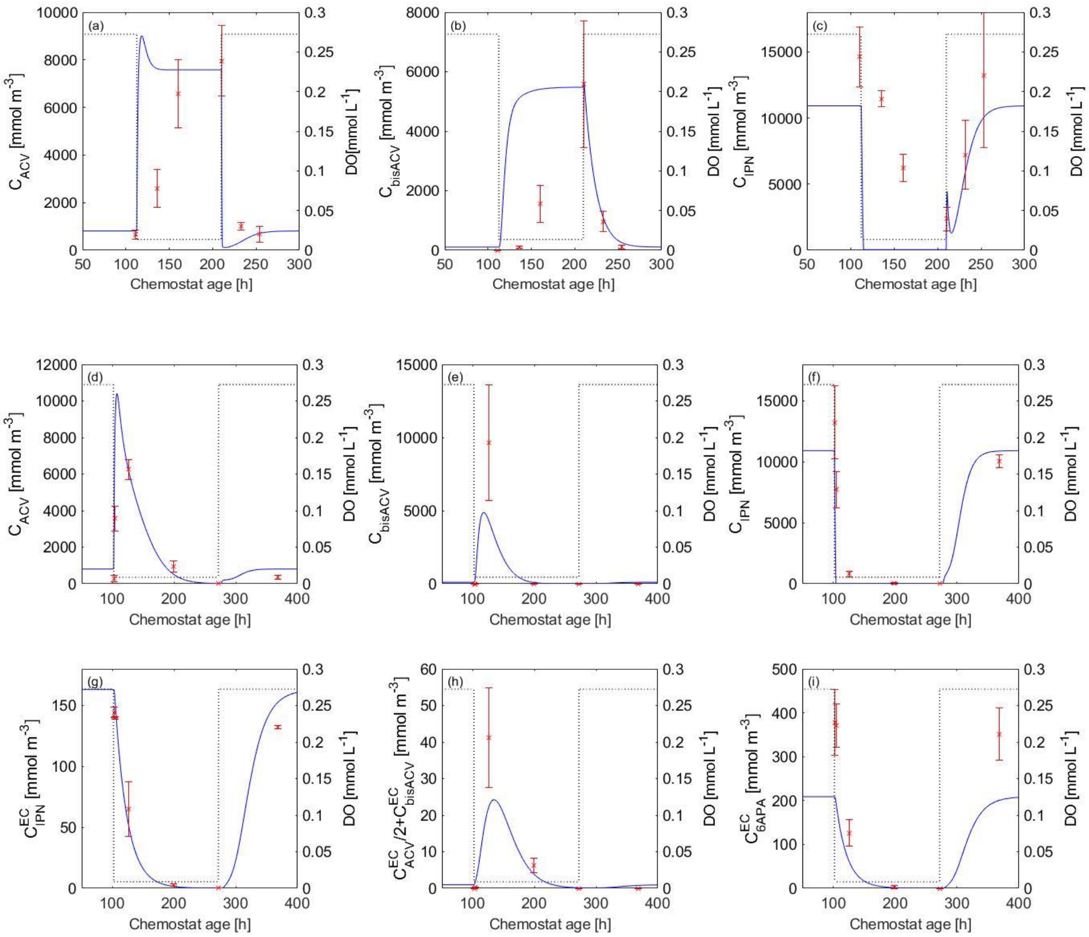

Figure B10. Measured (red crosses) and simulated (solid blue line) metabolite concentrations of the penicillin pathway (intracellular ACV, bisACV and IPN and extracellular IPN, bisACV and 6APA). The dotted line shows the simulated DO profile. a)-c): intracellular metabolite levels during the 0.013 mmol L^{-1} step experiment, d)-f) intracellular metabolite levels during the 0.009 mmol L^{-1} step experiment, g)-i) extracellular metabolite levels during the 0.009 mmol L^{-1} step experiment. The measured data points are calculated as averages between the duplicate runs. The error bar represents the standard deviation corresponding to multiple measurements and the duplicate runs. The predicted extracellular bisACV was calculated as the sum of the extracellular bisACV concentration and ACV concentration divided by two.

Simulation of the oscillation experiments

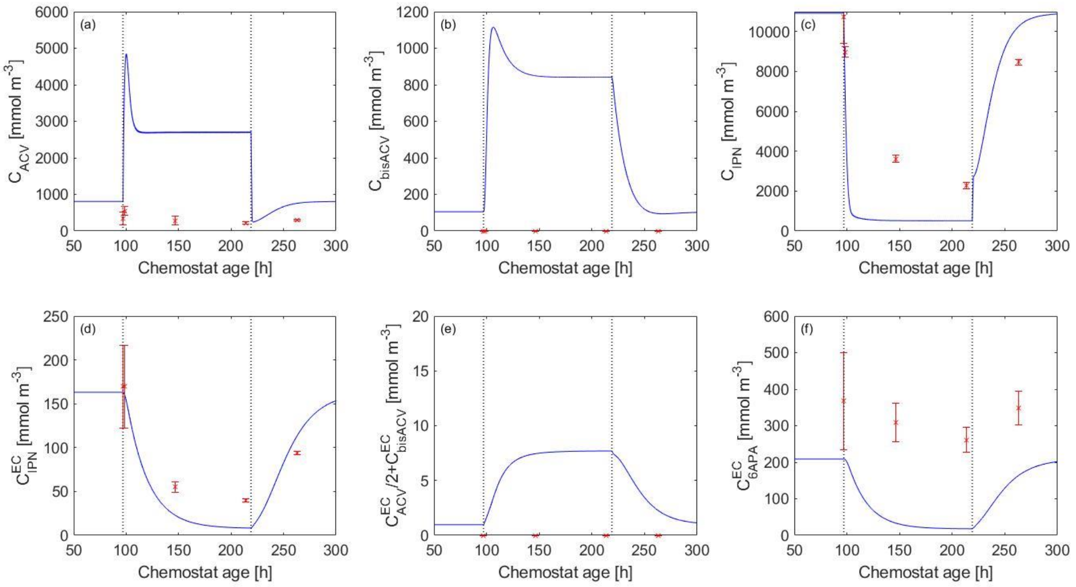

Figure B11. Measured (red crosses) and simulated (solid blue line) metabolite levels (intracellular ACV, bisACV and IPN concentrations, and extracellular IPN, bisACV and 6APA concentrations) during the oscillation experiment I. The vertical dotted lines represent the start and the end of the oscillation phase. The predicted extracellular bisACV was calculated as the sum of the extracellular bisACV concentration and ACV concentration divided by two.

DO profile oscillation experiments

The DO probe signal was corrected for the delayed response time of the probes to obtain the actual DO. The measured probe time constant was 18.34±2.35 seconds during the oscillation experiment I for the conventional DO probes, and 10.78±2.05 seconds for the optical DO probe used during the oscillation experiment II.

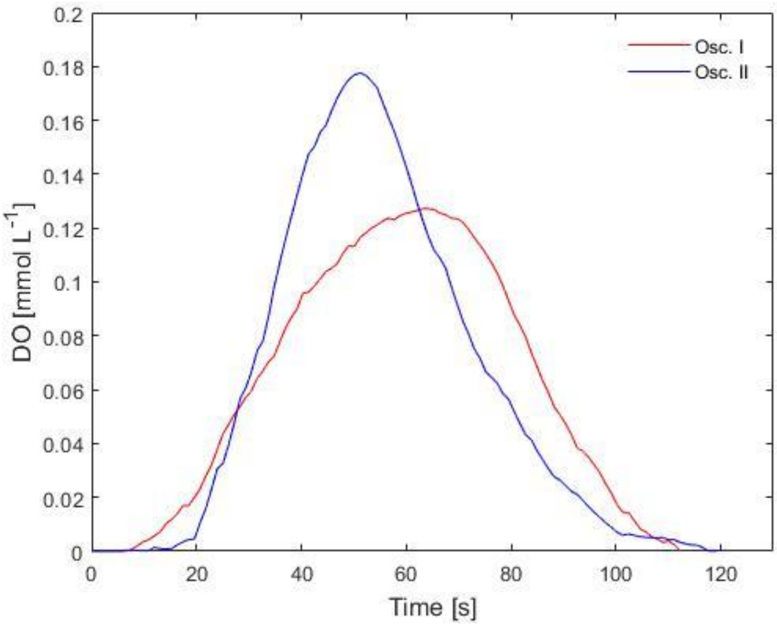

Figure B12. DO profile during the oscillation experiments. The DO was calculated taking into account the probe delay and a 9s moving average was applied to smoothen the curves.

Model simulations on second scale during the oscillation experiments

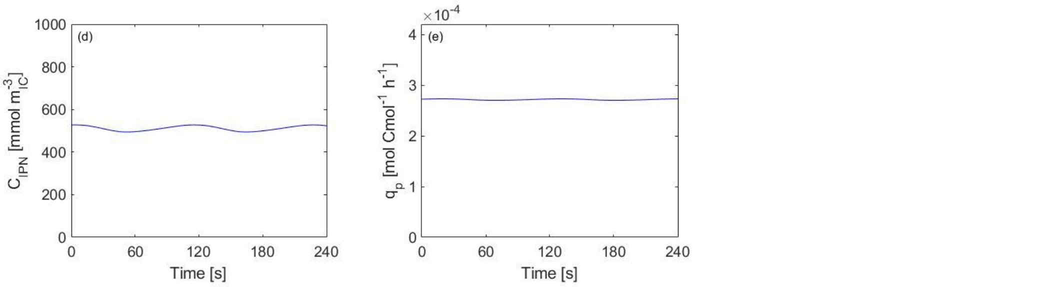

Figure B13. a) Experimentally obtained DO as model input, and predicted C_s (b), IPNS conversion rate, v_{IPNS} (c), IPN concentration (d) and q_p (e) during the oscillation experiment I. The results are plotted on a second scale, representing changes within the oscillation cycles.

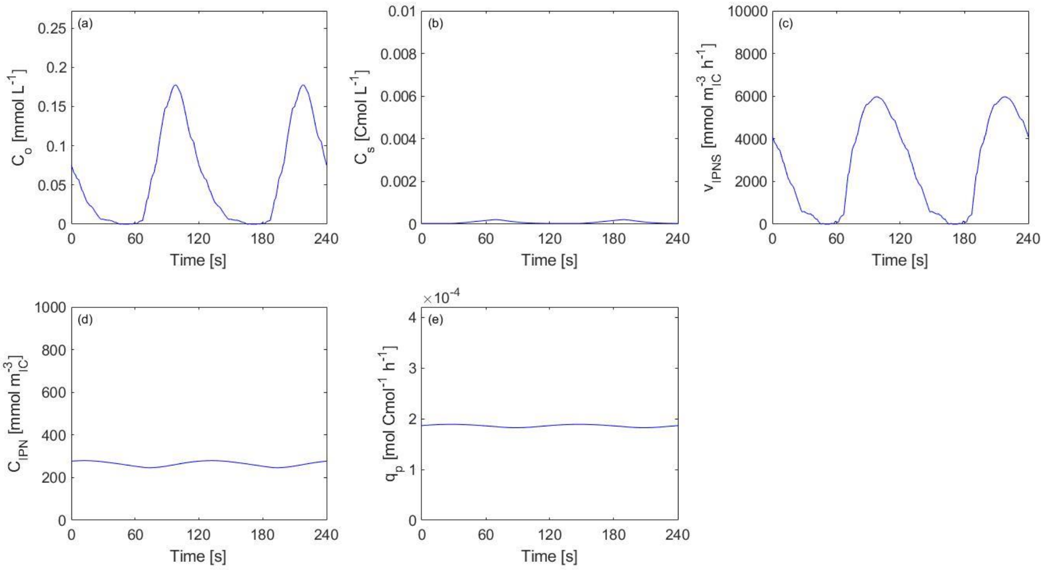

Figure B14. a) Experimentally obtained DO as model input, and predicted C_s (b), IPNS conversion rate, v_{IPNS} (c), IPN concentration (d) and q_p (e) during the oscillation experiment II. The results are plotted on a second scale, representing changes withing the oscillation cycles.

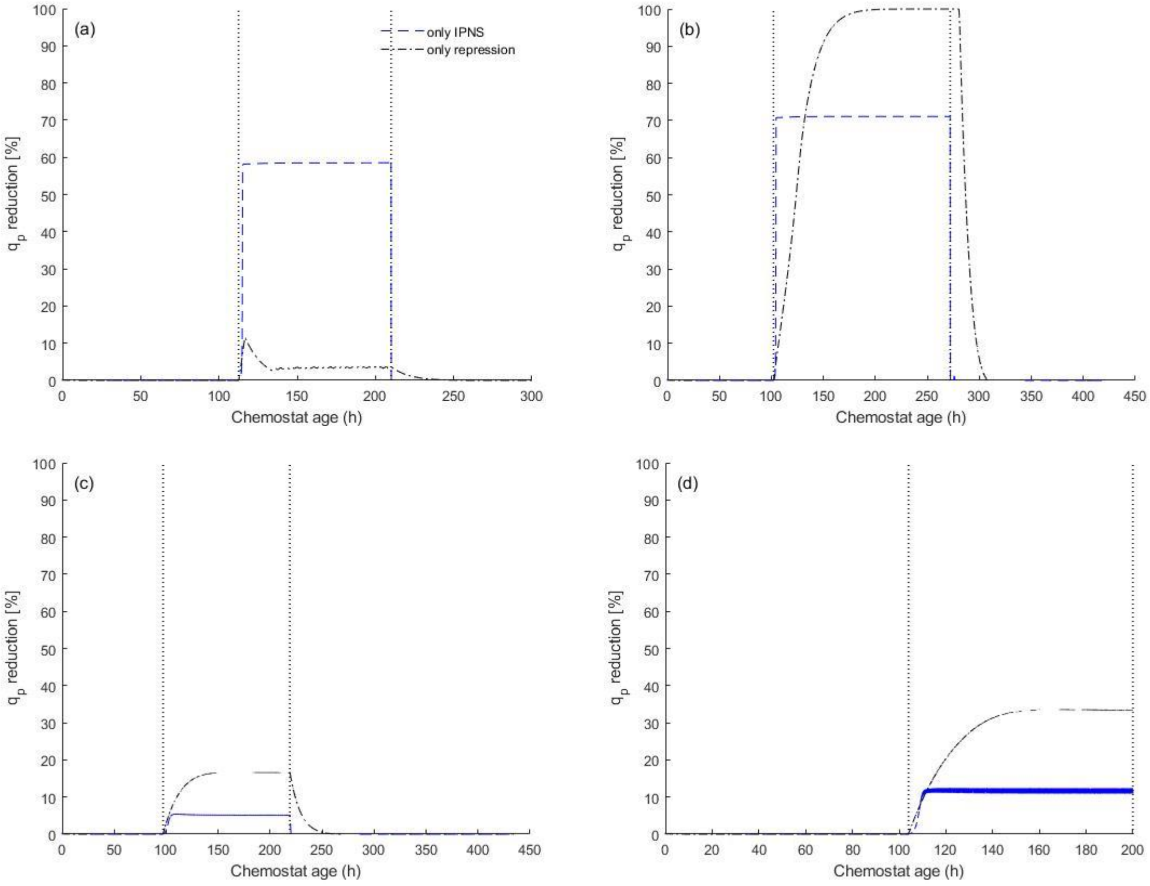

Figure B15. Reduction in the penicillin production rate when only the effect of O_2 on the IPNS enzyme is considered, excluding the influence of the glucose repression; and the reduction in the penicillin production rate when only the effect of

glucose repression is considered excluding the influence of O_2 on the IPNS enzyme. a) 0.013 mmol L^{-1} DO step experiment, b) 0.009 mmol L^{-1} DO step experiment, c) oscillation experiment I, d) oscillation experiment II.

References

[1] Seifar, R.M., Deshmukh, A.T., Heijnen, J.J., Van Gulik, W.M., Determination of δ-[L-α-aminoadipyl]-L-cysteinyl-D-valine in cell extracts of Penicillium chrysogenum using ion pair-RP-UPLC-MS/MS. *J. Sep. Sci.* 2012, *35*, 225–230.

[2] Deshmukh, A.T., Elucidation and modeling of the in-vivo kinetics of enzymes and membrane transporters associated with β-lactam and non-ribosomal peptide production in Penicillium chrysogenum. 2013.

[3] Jørgensen, H., Nielsen, J., Villadsen, J., Møllgaard, H., Metabolic flux distributions in Penicillium chrysogenum during fed-batch cultivations. *Biotechnol. Bioeng.* 1995, *46*, 117–131.

[4] Rodrıguez-Sáiz, M., Barredo, J.L., Moreno, M.A., Fernandez-Canon, J.M., et al., Reduced function of a phenylacetate-oxidizing cytochrome p450 caused strong genetic improvement in early phylogeny of penicillin-producing strains. *J. Bacteriol.* 2001, *183*, 5465–5471.

[5] Douma, R.D., Verheijen, P.J.T., de Laat, W.T.A.M., Heijnen, J.J., et al., Dynamic gene expression regulation model for growth and penicillin production in Penicillium chrysogenum. *Biotechnol. Bioeng.* 2010, *106*, 608–618.

[6] Gutiérrez, S., Marcos, A.T., Casqueiro, J., Kosalková, K., et al., Transcription of the pcbAB, pcbC and penDE genes of Penicillium chrysogenum AS-P-78 is repressed by glucose and the repression is not reversed by alkaline pHs. *Microbiology* 1999, *145*, 317–324.

[7] Nielsen, J., Jorgensen, H.S., Metabolic control analysis of the penicillin biosynthetic pathway in a high-yielding strain of Penicillium chrysogenum. *Biotechnol. Prog.* 1995, *11*, 299–305.

[8] Jørgensen, H., Nielsen, J., Villadsen, J., Møllgaard, H., Analysis of penicillin V biosynthesis during fed-batch cultivations with a high-yielding strain of Penicillium chrysogenum. *Appl. Microbiol. Biotechnol.* 1995, *43*, 123–130.

[9] Henriksen, C.M., Nielsen, J., Villadsen, J., Cyclization of α-aminoadipic acid into the δ-lactam 6-oxo-piperidine-2-carboxylic acid by Penicillium chrysogenum. *J. Antibiot. (Tokyo).* 1998, *51*, 99–106.

[10] Deshmukh, A.T., Verheijen, P.J.T., Seifar, R.M., Heijnen, J.J., et al., In vivo kinetic analysis of the penicillin biosynthesis pathway using PAA stimulus response experiments. *Metab. Eng.* 2015, *32*, 155–173.
